# Pontin functions as an essential coactivator for Oct4-dependent lincRNA expression in mouse embryonic stem cells

**DOI:** 10.1038/ncomms7810

**Published:** 2015-04-10

**Authors:** Kyungjin Boo, Jinhyuk Bhin, Yoon Jeon, Joomyung Kim, Hi-Jai R. Shin, Jong-Eun Park, Kyeongkyu Kim, Chang Rok Kim, Hyonchol Jang, In-Hoo Kim, V. Narry Kim, Daehee Hwang, Ho Lee, Sung Hee Baek

**Affiliations:** 1 Creative Research Initiative Center for Chromatin Dynamics, School of Biological Sciences, Seoul National University, Seoul 151-742, South Korea; 2 Department of Chemical Engineering, POSTECH, Pohang 790-784, South Korea; 3 Research Institute, Graduate School of Cancer Science and Policy, National Cancer Center, Gyeonggi-do 410-769, South Korea; 4 Institute for Basic Science, School of Biological Sciences, Seoul National University, Seoul 151-742, South Korea; 5 Department of New Biology and Center for Plant Aging Research, Institute for Basic Science, DGIST, Daegu 711-873, South Korea

## Abstract

The actions of transcription factors, chromatin modifiers and noncoding RNAs are crucial for the programming of cell states. Although the importance of various epigenetic machineries for controlling pluripotency of embryonic stem (ES) cells has been previously studied, how chromatin modifiers cooperate with specific transcription factors still remains largely elusive. Here, we find that Pontin chromatin remodelling factor plays an essential role as a coactivator for Oct4 for maintenance of pluripotency in mouse ES cells. Genome-wide analyses reveal that Pontin and Oct4 share a substantial set of target genes involved in ES cell maintenance. Intriguingly, we find that the Oct4-dependent coactivator function of Pontin extends to the transcription of large intergenic noncoding RNAs (lincRNAs) and in particular linc1253, a lineage programme repressing lincRNA, is a Pontin-dependent Oct4 target lincRNA. Together, our findings demonstrate that the Oct4-Pontin module plays critical roles in the regulation of genes involved in ES cell fate determination.

## Abstract (web summary)

Long non-coding RNAs or lincRNAs identified in embryonic stem (ES) cells have been shown to regulate ES cell states; however, how these lincRNAs are regulated remains unclear. Here the authors show that the transcriptional coactivator Pontin regulates the expression of lincRNAs involved in ES cell maintenance in an Oct4-dependent manner.

## Graphical abstract

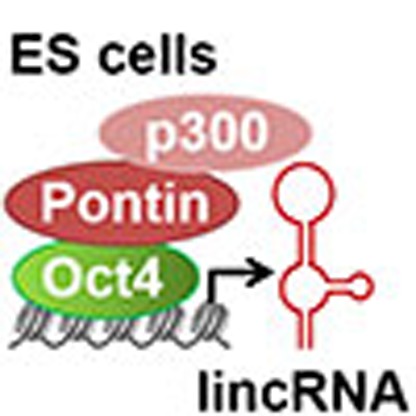

ES cells are derived from the inner cell mass of the mammalian embryos at the blastocyst stage and possess an unlimited potential for both self-renewal, the ability to proliferate without a change in phenotype, and pluripotency, the ability to differentiate into any cell in the organism^1^,^2^. ES cell-specific transcription factors, such as Oct4 and Nanog, have been identified as key factors for the formation and maintenance of the inner cell mass during development as well as self-renewal of ES cells^3^,^4^,^5^,^6^,^7^. In addition to the core transcription factors, a subset of chromatin modifiers has been implicated in pluripotency. The p300 histone acetyltransferase is predominantly recruited to Oct4-Nanog binding loci on mouse genome in ES cells^8^. Lack of p300 causes abnormal expression of germ layer markers during embryoid body (EB) formation from ES cells^9^. Depletion of Tip60 histone acetyltransferase complex, such as Tip60, p400 and Dmap1, causes loss of ES cell identity including reduction of S phase in cell cycle, weakened alkaline phosphatase (AP) activities and abnormal morphology^10^.

Recent studies have highlighted that non-coding RNAs are important for both the maintenance of pluripotency and repression of differentiation programme in ES cells, along with key transcription factors and chromatin modifiers^11^,^12^,^13^. LincRNAs are a subclass of long non-coding RNAs and have multi-exons and poly-A-tails like messenger RNAs^14^,^15^. LincRNAs have been shown to act in the circuitry controlling pluripotency and differentiation of ES cells^16^,^17^. There are over 200 lincRNAs identified in ES cells, and some of them including *linc1368*, *linc1577* and *linc1634* function to maintain the pluripotent state through regulation of Oct4 or Nanog expression^18^. The majority of lincRNAs expressed in ES cells are also targets of ES cell-specific transcription factors. Therefore, lincRNAs may function as downstream targets and/or upstream regulators of these transcription factors or chromatin modifiers^19^,^20^,^21^. Despite the critical roles of lincRNAs in ES cells, molecular mechanisms for the regulation of lincRNA expression have not yet been extensively studied.

Proper regulation of chromatin structure by the coordinated action of transcription factors and chromatin modifiers is important for cell state-specific gene expression^22^,^23^,^24^,^25^,^26^,^27^. Pontin is a chromatin remodelling factor that possesses both ATPase and DNA helicase activities^28^,^29^,^30^. Pontin functions as a coactivator for various transcription factors including androgen receptor (AR) in prostate cancer, T-cell factor (TCF) in the Wnt signalling pathway and hypoxia-inducible factor-1α (HIF-1α) in the hypoxia signalling pathway^31^,^32^,^33^. It has been shown that Tip60 histone acetyltransferase complex possesses Pontin as a component in addition to p400^34^,^35^. An RNAi screen of chromatin proteins identified Tip60-p400 as a critical regulator complex of ES cell identity. Further, chromatin remodelling complexes and polycomb group proteins are implicated in ES cell maintenance^36^,^37^,^38^,^39^. Although these reports provide clues for the importance of chromatin remodelling complexes, the underlying mechanisms remain largely unknown.

Here, we report that Pontin deficiency in ES cells severely compromises ES cell maintenance and Pontin functions as a critical coactivator for Oct4. ChIP-sequencing and mRNA-sequencing analyses identify a substantial amount of overlapping target genes between Oct4 and Pontin in ES cells. Intriguingly, a subset of Oct4-dependent lincRNAs is regulated by Oct4-Pontin module, and these Oct4/Pontin-dependent lincRNAs are mainly involved in the repression of differentiation programme in ES cells. Together, these findings demonstrate a functional link between lincRNAs and chromatin modifiers, which is mediated by Oct4 to orchestrate the programming of cell states in ES cells.

## Results

### Targeted disruption of *Pontin* causes defects in ES cell maintenance

To explore the biological function of Pontin *in vivo*, we generated *Pontin*-deficient mice by gene targeting in ES cells (Supplementary Fig. 1a-c). Although *Pontin* heterozygous (*Pontin*^+/−^) mice were fertile and showed no detectable developmental abnormalities over 1.5-year-observation period, none of the *Pontin*-deficient homozygous (*Pontin*^−/−^) animals were obtained from 168 offsprings (Supplementary Fig. 1d), indicating that lack of *Pontin* resulted in embryonic lethality. Analysis of *Pontin*^−/−^ embryos revealed that they were competent for pre-implantation development and died between E3.5 and E7.5 (Supplementary Fig. 1d).

**Figure 1:**
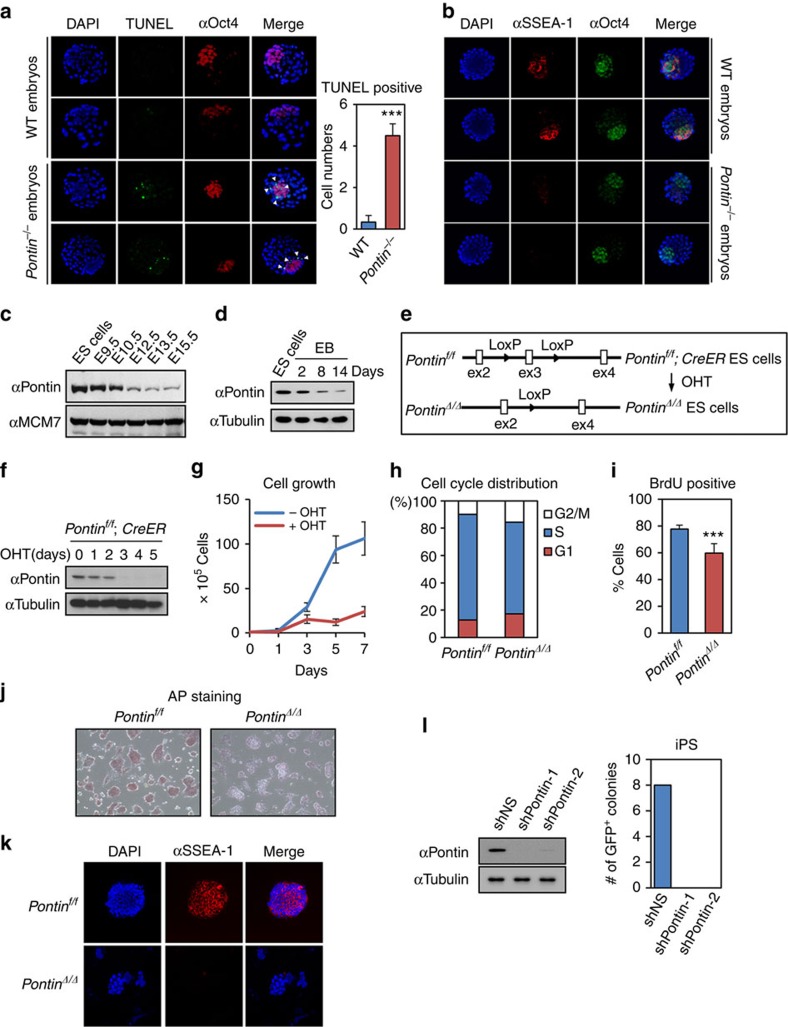
*Pontin* deficiency causes the defects in mouse ES cell maintenance. (**a**) WT and *Pontin*-deficient E5.0 embryos were stained using the TUNEL assay system. Apoptotic cells were shown by TUNEL (green) and nuclei were stained with DAPI (blue). The epiblast cells were immunostained with an anti-Oct4 antibody (red). Representative images are shown. Right side histogram represents the numbers of TUNEL-positive cells per embryo (mean±s.d.). *P* value was calculated by *t*-test (*n*=10 for each group; ***P=2.03 × 10^−8^). Magnification × 40. (**b**) Representative images show decreased SSEA-1 expression (red) in inner cell mass of *Pontin*-deficient embryos (*n*=4 for each group). (**c**,**d**) Reduction of Pontin expression during mouse embryo development stages (**c**) and EB differentiation (**d**). (**e**) Diagram of the strategy for depletion of *Pontin* using *Pontin*^*f/f*^; *CreER* ES cells. (**f**) Depletion of *Pontin* in *Pontin*^*f/f*^; *CreER* ES cells at indicated days after OHT treatment. (**g**) Growth curves of *Pontin*^*f/f*^; *CreER* ES cells in the absence or presence of OHT. These experiments were independently repeated three times. (**h**) Cell cycle-phase analysis of *Pontin*^*f/f*^; *CreER* ES cells in the absence or presence of OHT. Cells were harvested at 3 days after treatment with vehicle (95% EtOH) or OHT. Similar results were obtained from three independent experiments. (**i**) BrdU incorporation was used to determine the proportion of the cells in S phase. WT or *Pontin*-depleted ES cells were harvested at 3 days after vehicle or OHT treatment. The percentages of BrdU positive cells are graphed. Values are expressed as mean±s.d. of three independent experiments. ***P<0.001. (**j**) Representative images show the reduction of AP activity of *Pontin*-depleted ES cells compared with WT. Cells were stained at 3 days after vehicle or OHT treatment. Similar results were obtained from three independent experiments. Magnification × 10. (**k**) SSEA-1 expression in *Pontin*-depleted ES cells. Colonies of undifferentiated WT and *Pontin*-depleted ES cells were stained as indicated. Representative images are shown. Similar results were obtained from three independent experiments. Magnification × 20. (**l**) Knockdown of Pontin reduces reprogramming efficiency. The number of GFP-positive colonies was presented.

We next examined the roles of Pontin in both survival and maintenance of pluripotent cells in inner cell mass. A TUNEL assay, with embryos from heterozygote intercrosses at the blastocyst stage, showed that *Pontin*-deficient embryos exhibited increased apoptotic cell numbers, whereas WT and heterozygous littermates exhibited little or no apoptotic cells in inner cell mass (Fig. 1a). Further, *Pontin* deficiency reduced expression of stage-specific embryonal antigen-1 (SSEA-1), a marker for murine pluripotent stem cells (Fig. 1b). Pontin expression was significantly reduced during both embryogenesis (Fig. 1c) and *in vitro* differentiation process after EB formation (Fig. 1d).

We generated *Pontin*^*f/f*^; *CreER* ES cells, in which *Pontin* can be conditionally deleted by 4-hydroxy tamoxifen (OHT) treatment (Fig. 1e and Supplementary Fig. 1e). Protein levels of Pontin were almost completely depleted after 3 days of OHT administration (Fig. 1f and Supplementary Fig. 1f). To examine whether *Pontin* depletion affects ES cell growth, we counted cell numbers over several days. The growth of *Pontin*-depleted ES cells was significantly reduced compared with that of WT (Fig. 1g). Propidium iodide staining of *Pontin*-depleted ES cells followed by fluorescence-activated cell sorter (FACS) analysis of cell cycle-phase distribution revealed a reduction of S-phase cells (Fig. 1h). A BrdU incorporation experiment showed a reduction of BrdU-positive cells in *Pontin*-depleted ES cells (Fig. 1i). Further, we measured AP activity, which is high in undifferentiated ES cells but significantly decreased during differentiation. *Pontin*-depleted ES cells showed weak AP staining with loss of ES cell morphology (Fig. 1j). SSEA-1 expression was significantly reduced in *Pontin*-depleted ES cells (Fig. 1k). Together, these data indicate that depletion of *Pontin* leads to the defects in ES cell maintenance. We also examined the role of Pontin in reprogramming of fibroblasts to induced pluripotent cells (iPSCs). Transduction of *Oct4*-promoter-driven-GFP MEFs (pOct4-GFP MEFs) with OSK (Oct4, Sox2 and Klf4) transgenes induced colonies with ESC colony-like morphology, and some of them expressed GFP, a marker of endogenous Oct4 expression. Knockdown of Pontin by shRNA reduced reprogramming efficiency (Fig. 1l), indicating that Pontin is important for somatic cell reprogramming. However, overexpression of Pontin did not affect iPSC formation efficiency significantly (Supplementary Fig. 1g).

### Identification of target genes regulated by Pontin in ES cells

**Figure 2:**
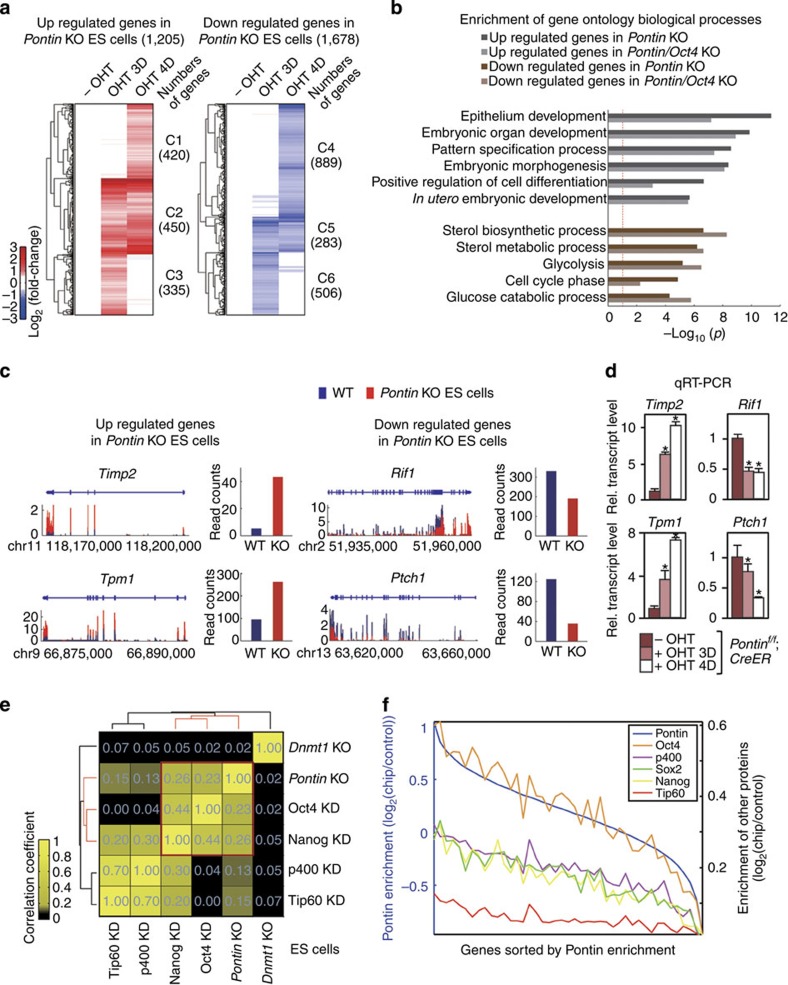
Identification of target genes regulated by Pontin in mouse ES cells. (**a**) Up- and downregulated genes in *Pontin*-depleted ES cells in comparison with *Pontin*^*f/f*^; *CreER* ES cells at 3 days (OHT 3D) or 4 days (OHT 4D) versus 0 day post-OHT treatment (-OHT). Hierarchical clustering identified six clusters of the up- (C1-C3) and downregulated (C4-C6) genes. Numbers of DEGs in the clusters are denoted in parenthesis. The colour bar represents the gradient of log_2_-fold-changes in each comparison. (**b**) Gene Ontology Biological Processes (GOBPs) represented by the up- and downregulated genes by *Pontin* depletion (dark bars) and by both *Pontin* depletion and *Oct4* depletion (light bars). The bars for GOBPs represent the enrichment scores, −log_10_(*p*), where *p* is *P* value that the GOBPs are enriched. The red line denotes the cutoff, *P*=0.1. (**c**) mRNA-sequencing reads of two upregulated genes, *Timp2* and *Tpm1*, and two downregulated genes, *Rif1* and *Ptch1*, in *Pontin*-depleted ES cells. Red and blue bars (*y* axis) along the genomic coordinate (*x* axis) represent read coverages for individual bases of the genes measured at OHT 4D from *Pontin*-depleted and WT ES cells, respectively. Each bar graph shows the normalized read counts of the corresponding gene in WT and *Pontin*-depleted ES cells. (**d**) Quantitative RT-PCR analysis of up- and downregulated genes in *Pontin*-depleted ES cells. The mRNA quantity was normalized by using primers to detect *Gapdh*. Error bars represent mean±s.d.; *P<0.05. (**e**) Comparison of ES cell expression profiles on gene depletion (KO) or knockdown (KD) of the indicated factors using hierarchical clustering of Pearson correlation coefficients of log_2_-fold-changes in KO or KD ES cells, compared with WT ES cells. Changes of gene expression in *Dnmt1*-depleted ES cells were used as the negative control. (**f**) Comparisons of binding enrichments of Pontin with those of Oct4, Sox2, Nanog, Tip60 and p400 from ChIP-sequencing analysis. *X* axis indicates the sorted genes by Pontin enrichment ratios, log_2_(Pontin-ChIP/GFP). Left *y* axis indicates the mean enrichment ratios of Pontin in individual bins of the sorted genes (500 genes/bin), and right *y* axis indicates the mean enrichment ratios of the other factors for the genes in the individual bins defined by Pontin.

To gain insights into the underlying mechanisms by which Pontin contributes to the ES cell maintenance, we performed genome-wide mRNA-sequencing analysis of *Pontin*^*f/f*^*; CreER* ES cells at 0, 3 or 4 days post-treatment with OHT (Fig. 2a). On average, 55.4 million reads were obtained in individual samples and aligned to the mouse genome, resulting in 5.3 Giga bps of mapped sequences, which corresponds to 52.2-fold coverage of the annotated mouse transcriptome (Supplementary Data 1). To explore the downstream target genes of Pontin, we compared gene expression in *Pontin*-depleted ES cells relative to that of WT and identified differentially expressed genes (DEGs) that consist of 1,205 upregulated and 1,678 downregulated genes in *Pontin*-depleted ES cells compared with WT (Fig. 2a). The cellular processes affected by Pontin were then examined by performing functional enrichment analysis of the DEGs using DAVID software^40^ (Fig. 2b and Supplementary Data 2). This analysis indicates that the majority of upregulated genes in *Pontin*-depleted ES cells are mainly related to differentiation and embryonic developmental processes, whereas downregulated genes are involved in cell cycle and metabolism, which are essential for ES cell maintenance (Fig. 2b). Consistent with this observation, the genes related to development and differentiation (that is, *Timp2* and *Tpm1*) are upregulated in *Pontin*-depleted ES cells, whereas the genes involved in stem cell maintenance and metabolism (that is, *Rif1* and *Ptch1*) are downregulated as evidenced by mRNA sequencing (Fig. 2c) and quantitative RT-PCR analyses (Fig. 2d).

To understand the association of Pontin with ES-specific transcription factors and/or chromatin modifiers in transcriptional programme, we compared the mRNA-sequencing data of *Pontin*-depleted ES cells with previously reported gene expression profiles of ES cells deficient of ES cell-specific transcription factors such as Oct4 and Nanog, and chromatin modifiers such as Tip60 and p400, which form a histone acetyltransferase complex with Pontin. For the comparison, we evaluated pair-wise correlations of the overall changes of gene expression in *Pontin*-depleted ES cells with those in ES cells deficient of the other factors. Interestingly, the analysis revealed that Pontin showed more significant correlation with Oct4 or Nanog than Tip60 or p400 (Fig. 2e). The strong correlations of Pontin with Oct4 or Nanog were confirmed by an independent correlation analysis (Supplementary Fig. 2a). Further, we performed ChIP-sequencing using an anti-Pontin antibody and compared the data with those previously generated with anti-Oct4, Sox2, Nanog, Tip60 and p400 antibodies in mouse ES cells using correlation analysis of binding enrichment in the promoter and enhancer regions^8^,^41^ (Fig. 2f and Supplementary Data 1). Consistent with the above analysis, this correlation analysis revealed that Pontin exhibited more significant correlation with Oct4 than Sox2, Nanog, p400 and Tip60. These strong correlations between Pontin and Oct4 in both mRNA-sequencing and ChIP-sequencing data indicate that Pontin-Oct4 may regulate a large number of common target genes cooperatively.

### Pontin and Oct4 share a substantial number of target genes in ES cells

The unexpected observation of the significant overlap between the expression profiles of *Pontin-*depleted ES cells and *Oct4*-depleted ES cells led us to further explore the functional link between Pontin and Oct4. To search for their shared target genes, we further performed mRNA sequencing of *Oct4*-depleted ES cells generated from ZHBTc4 cells, a conditional *Oct4*-depleted ES cell line, in which Oct4 can be depleted by tetracycline treatment for 2 days^42^. The mRNA-sequencing analysis of *Oct4*-depleted ES cells showed that 55.2 million reads were aligned to the mouse genome, resulting in 5.2 Giga bps of mapped sequences, which corresponds to 54.1-fold coverage of the annotated mouse transcriptome (Supplementary Data 1). Similar to the case of *Pontin*-depleted ES cells, most of upregulated genes in *Oct4*-depleted ES cells are involved in differentiation and developmental processes, whereas downregulated genes are involved in self-renewal and metabolism (Supplementary Data 2).

**Figure 3:**
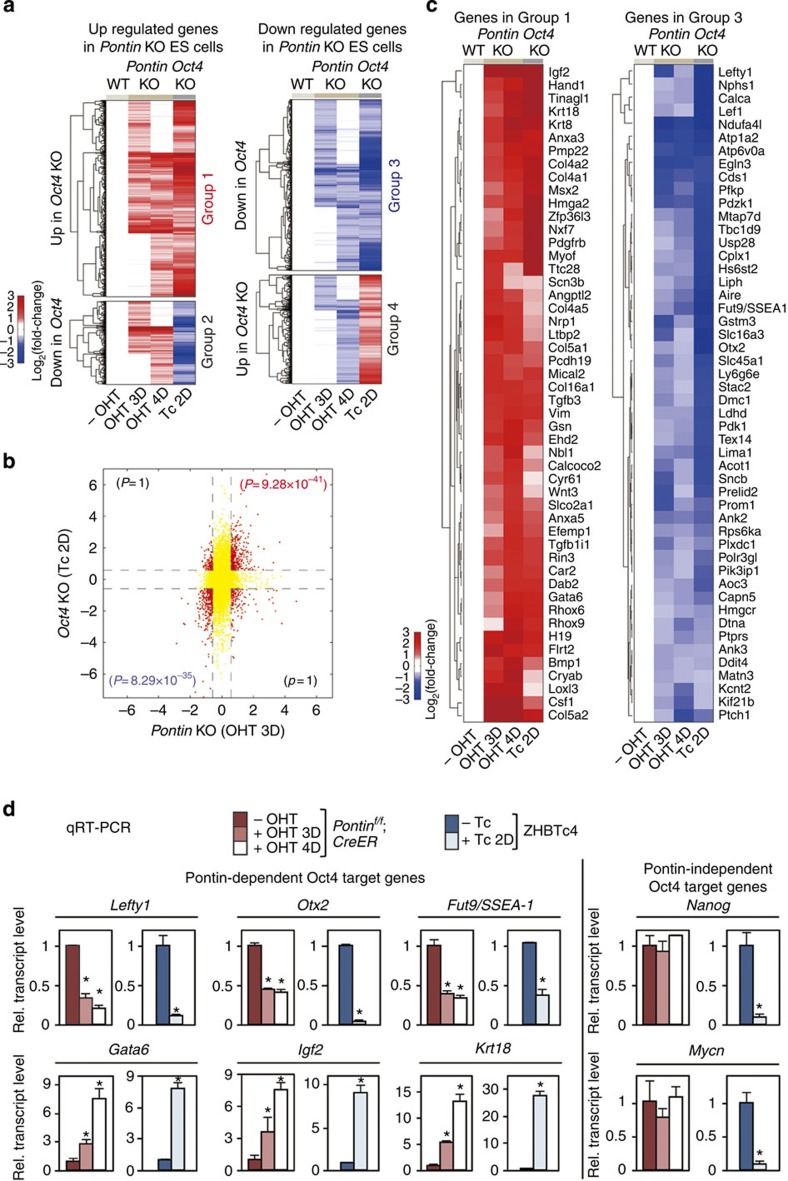
Comparative analyses of *Oct4*- and *Pontin*-depleted mouse ES cells. (**a**) The genes commonly regulated by Pontin and Oct4: Group 1, upregulated by both *Pontin* and *Oct4* depletion; Group 2, upregulated by *Pontin* depletion, but downregulated by *Oct4* depletion; Group 3, downregulated by both *Pontin* and *Oct4* depletion; Group 4, downregulated by *Pontin* depletion, but upregulated by *Oct4* depletion. *Pontin*-depleted ES cells are *Pontin*^*f/f*^; *CreER* ES cells at 3 days (OHT 3D) or 4 days (OHT 4D) post-OHT treatment. Depletion of Oct4 is generated from ZHBTc4 ES cells by treatment of tetracycline for 2 days (Tc 2D). No OHT-treated *Pontin*^*f/f*^; *CreER* ES cells are used as counterpart WT ES cells (-OHT). Hierarchical clustering of log_2_-fold-changes from the comparisons of *Pontin*-depleted and *Oct4*-depleted ES cells versus WT ES cells is used to display the up- (red) and downregulated (blue) genes. The colour bar represents the gradient of log_2_-fold-changes in each comparison. (**b**) Comparison of the log_2_-fold-changes between *Pontin*-depleted (OHT 3D) and *Oct4*-depleted (Tc 2D) ES cells. In the scatter plot, the red dots represent the DEGs (≥ 1.5-fold) in the comparisons of *Pontin*-depleted ES cells versus WT and *Oct4*-depleted ES cells versus WT. The significance for the number of genes in each quadrant was computed by Fisher's exact test. (**c**) Top 50 shared up- (left panel) and downregulated (right panel) genes by *Pontin* depletion and *Oct4* depletion. (**d**) Quantitative RT-PCR analysis showing mRNA levels of Oct4 target genes, which are Pontin-dependent or Pontin-independent in ES cells depleted with *Pontin* or *Oct4*. The quantity of mRNA was normalized by using primers to detect *Gapdh*. Values are expressed as mean±s.d. of three independent experiments. *P<0.05.

We compared the DEGs in *Pontin*-depleted ES cells and *Oct4*-depleted ES cells (Fig. 3a and Supplementary Data 3). Among 1,205 genes upregulated in *Pontin*-depleted ES cells, 744 genes are regulated by Oct4 (Groups 1 and 2 in Fig. 3a), and out of 1,678 genes that are downregulated in *Pontin*-depleted ES cells, 912 genes are regulated by Oct4 (Groups 3 and 4 in Fig. 3a). Interestingly, the majority of the upregulated genes by *Pontin* depletion are also upregulated by *Oct4* depletion (*P*=9.28 × 10^−41^ by Fisher's exact test) (Fig. 3b and Supplementary Fig. 2b; see Top 50 shared upregulated genes in Fig. 3c). The upregulated genes in Group 1 are mainly involved in differentiation and embryonic development processes (Supplementary Data 2). We confirmed that developmental genes such as *Gata6*, *Igf2* and *Krt18* are upregulated in *Pontin*-depleted ES cells and *Oct4*-depleted ES cells by quantitative RT-PCR analysis (Fig. 3d). Furthermore, we found a strong correlation among the downregulated genes in *Oct4*-depleted and *Pontin*-depleted ES cells (*P*=8.29 × 10^−35^ by Fisher's exact test) (Fig. 3b and Supplementary Fig. 2b; see Top 50 shared downregulated genes in Fig. 3c). The downregulated genes in Group 3 are closely related to cell cycle and metabolism, which are important for ES cell maintenance (Supplementary Data 2). Quantitative RT-PCR analysis confirmed that Oct4 target genes involved in self-renewal and ES cell maintenance, such as *Lefty1*, *Otx2* and *Fut9/SSEA-1,* are downregulated in *Oct4*-depleted ES cells and *Pontin*-depleted ES cells (Fig. 3d). Some Oct4 target genes such as *Nanog* and *Mycn* are not affected by Pontin. Together, these data indicate that Pontin and Oct4 share a substantial number of downstream target genes required for the ES cell maintenance.

To test the possibility that *Pontin* possesses Oct4 and/or Nanog binding sites in the promoter and enhancer regions, we examined binding sites of Oct4 and/or Nanog based on the previous binding site analysis of two sets of ChIP-sequencing data (GSE11431 and GSE11724)^43^. Both data sets predicted no binding sites of Oct4 or Nanog in the region between 5 kb-upstream and 1 kb-downstream from the transcription start site (TSS) of *Pontin* (Supplementary Fig. 3a). Furthermore, no significant differences of both protein and mRNA levels of Pontin were found by Oct4 or Nanog depletion in ES cells (Supplementary Fig. 3b,c), indicating that *Pontin* is not a direct target gene of Oct4 or Nanog.

### Pontin functions as a transcriptional coactivator for Oct4 in ES cells

**Figure 4:**
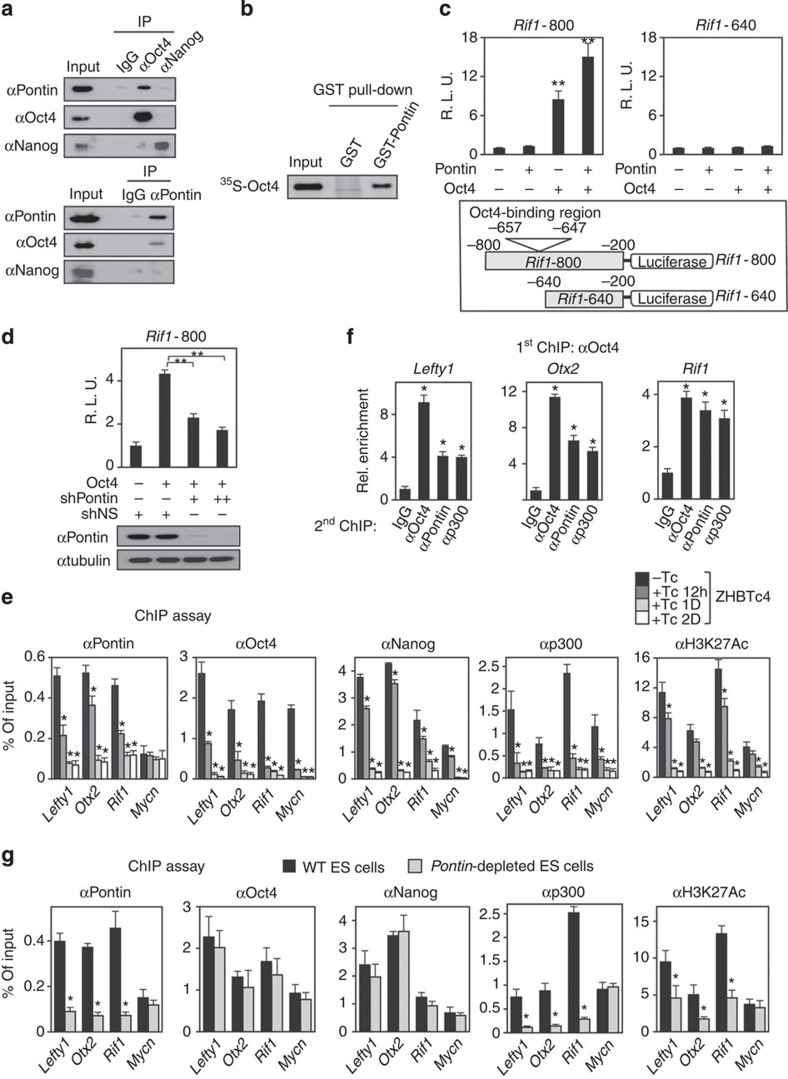
Pontin functions as a transcriptional coactivator for Oct4 in ES cells. (**a**) Specific association of Pontin with Oct4 at endogenous expression level in ES cells by reciprocal co-immunoprecipitation assay. Immunoblot analysis using antibodies specific for the indicated proteins from ES cells was shown. (**b**) GST pull-down assay was performed using *in vitro* translated ^35^*S*-methionine-labelled Oct4 with GST-Pontin. (**c**,**d**) Luciferase assay was performed with reporters driven by *Rif1* promoter possessing Oct4-binding elements (*Rif1*-800) or deleted Oct4-binding region (*Rif1*-640). Effect of Pontin expression on *Rif1*-800 or *Rif1*-640 promoter-luciferase activity in the presence of Oct4 (**c**) or effect of *Pontin* knockdown on *Rif1*-800 promoter luciferase activity (**d**) was shown. Luciferase activities were normalized by β-galactosidase activity. Values are expressed as mean±s.d. of three independent experiments. **P<0.01. (**e**) ChIP assays were performed on enhancers or promoters of Pontin-dependent or -independent Oct4 target genes with indicated antibodies in ZHBTc4 ES cells after vehicle or tetracycline treatment for indicated times. Values are expressed as mean±s.d. of three independent experiments. *P<0.05. (**f**) Two-step ChIP assays were performed in ZHBTc4 ES cells. The chromatin fractions were first subject to pull-down with anti-Oct4 antibodies, eluted from immunocomplexes and applied for the second pull-down with control IgG or the reciprocal antibodies. Values are expressed as mean±s.d. of three independent experiments. *P<0.05. (**g**) ChIP assays were performed on enhancers or promoters of Pontin-dependent or -independent Oct4 target genes with indicated antibodies in WT and *Pontin*-depleted ES cells. Values are expressed as mean±s.d. of three independent experiments. *p<0.05.

As Pontin has been shown as a coactivator for many transcription factors, we checked the possibility that Pontin functions as a coactivator for Oct4 and forms a specific regulatory module for downstream target genes. First, we examined mutual binding of Pontin and Oct4 or Nanog by co-immunoprecipitation assay and found that endogenous Pontin binds to Oct4, but not Nanog, in ES cells (Fig. 4a). GST pull-down assay confirmed direct interaction of Pontin with Oct4 (Fig. 4b). Next, we performed a luciferase assay with reporters driven by *Rif1* promoter possessing functional Oct4 binding elements, based on the identification of *Rif1* as one of Pontin target genes from our mRNA-sequencing analysis. We constructed two different luciferase reporter plasmids, one containing Oct4-binding region (*Rif1*-800) and the other containing deleted Oct4-binding region (*Rif1*-640). Introduction of Oct4 increased *Rif1*-800 promoter-luciferase activity, and overexpression of Pontin potentiated Oct4-dependent activation of *Rif1*-800 promoter-luciferase reporter, but not *Rif1*-640 promoter-luciferase reporter (Fig. 4c), indicating that Pontin functions as a coactivator for Oct4. Further, Oct4-mediated increase of *Rif1*-800 promoter-luciferase activity was attenuated by knockdown of Pontin (Fig. 4d).

We then performed ChIP assays in *Oct4*-depleted ES cells compared with WT ES cells to determine whether Pontin recruitment is Oct4-dependent. We examined Pontin-dependent Oct4 target genes such as *Lefty1*, *Otx2* and *Rif1*, and Pontin-independent Oct4 target gene such as *Mycn* for comparison. Consistent with previous reports^44^, Oct4 and p300 were co-recruited to Oct4-binding sites, concomitant with strong acetylation signal at lysine 27 of histone H3 (H3K27) in WT ES cells (Fig. 4e). Pontin was also recruited to the Oct4-binding regions of *Lefty1*, *Otx2* and *Rif1*, but not that of *Mycn*. However, on Oct4 depletion, recruitment of Pontin and p300 was significantly diminished to the Oct4-binding regions concomitant with the decreased H3K27 acetylation level (Fig. 4e). We performed two-step ChIP assays to further examine the Oct4 dependency of Pontin and p300 for the target promoter binding. The elutes from the first immunoprecipitation reaction with an anti-Oct4 antibody were re-immunoprecipitated with anti-Pontin or anti-p300 antibody, and the two-step ChIP assays revealed that the recruitment of Pontin and p300 on the target promoter is Oct4-dependent (Fig. 4f).

We further examined whether Pontin collaborates with p300 for Oct4-dependent transcriptional activation. First, we examined whether Pontin binds to p300 at endogenous expression level. Co-immunoprecipitation assay revealed that Pontin bound to p300 in ES cells (Supplementary Fig. 3d). Introduction of p300 increased *Rif1*-800 promoter-luciferase activity by Oct4 and Pontin, and the increase of *Rif1*-800 promoter-luciferase activity by Oct4 and Pontin was attenuated by knockdown of p300 (Supplementary Fig. 3e, f). Next, we compared recruitment of p300 along with H3K27 acetylation levels by ChIP assays in *Pontin*-depleted ES cells. Intriguingly, ChIP assays revealed that lack of Pontin led to the failure of p300 recruitment along with reduced H3K27 acetylation levels on the Pontin-Oct4 target genes such as *Lefty1*, *Otx2* and *Rif1*, but not on *Mycn* (Fig. 4g). Recruitment of neither Oct4 nor Nanog was affected by Pontin depletion. These results demonstrate that Pontin requires Oct4 binding for its recruitment to the Oct4-Pontin target promoters and collaborates with p300 to exert transcriptional coactivator function.

### Pontin functions as a coactivator for Oct4-dependent lincRNA transcription

**Figure 5:**
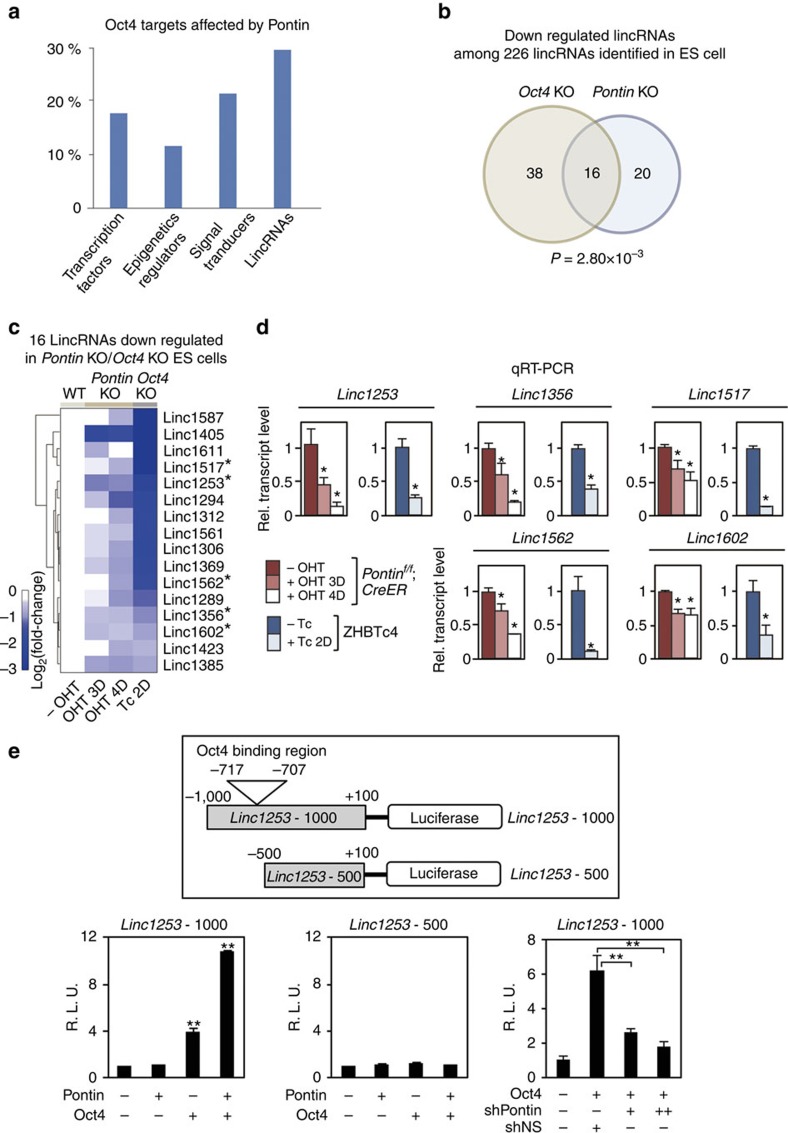
Pontin is required for transcription of a subset of lincRNAs regulated by Oct4. (**a**) Proportions of the downregulated genes in *Pontin*-depleted ES cells out of those in *Oct4*-depleted ES cells. (**b**) Venn diagram of the downregulated lincRNAs in *Oct4*-depleted and *Pontin*-depleted ES cells. Sixteen lincRNAs are downregulated in both *Pontin*-depleted and *Oct4*-depleted ES cells. (**c**) List of 16 lincRNAs that are downregulated in both *Oct4*-depleted and *Pontin*-depleted ES cells. Asterisk (*) denotes the lincRNAs previously reported to function in repression of lineage specification process in ES cells. (**d**) Quantitative RT-PCR analysis of five lincRNAs involved in repression of differentiation processes among 16 lincRNAs that are downregulated in both *Pontin*-depleted and *Oct4*-depleted ES cells. The quantity of lincRNA was normalized by using primers to detect *Gapdh*. Values are expressed as mean±s.d. of three independent experiments. *P<0.05. (**e**) Luciferase assay was performed with reporters driven by *linc1253* promoter possessing Oct4-binding elements (*linc1253*-1000) or deleted Oct4-binding region (*linc1253*-500). Luciferase activities were normalized by β-galactosidase activity. Values are expressed as mean±s.d. of three independent experiments. **p<0.01.

Chromatin signature mapping and global gene expression analysis with RNAi screening in ES cells showed that lincRNAs play a role in the maintenance of self-renewal and pluripotency. As a subset of lincRNAs has been shown to be directly regulated by Oct4, we tested the possibility that Pontin regulates expression of Oct4-dependent lincRNAs. Our analysis of Oct4 targets affected by Pontin revealed that lincRNAs are significantly affected by Pontin expression (Fig. 5a). Among the known 226 lincRNAs expressed in ES cells, 54 lincRNAs are downregulated in *Oct4*-depleted ES cells and 16 (30% in Fig. 5a, *P*=2.80 × 10^−3^ by Fisher's exact test) of the 54 lincRNAs are also downregulated in *Pontin*-depleted ES cells (Fig. 5b and Supplementary Data 4). Interestingly, five lincRNAs (*linc1253*, *linc1356*, *linc1517*, *linc1562* and *linc1602*) that are co-regulated by both Pontin and Oct4 from our analysis (denoted with asterisk in Fig. 5c) have been shown to function as repressors of lineage differentiation programme in ES cells^18^. Moreover, the genes that are upregulated by knockdown of *linc1253*, *linc1356* or *linc1517* significantly overlapped with those that are upregulated by *Pontin* depletion (false discovery rate<0.1) (Supplementary Fig. 4a). These analyses support that Pontin and lincRNAs may collaborate to repress lineage specification programmes in ES cells. Quantitative RT-PCR analysis confirmed that lack of either Oct4 or Pontin resulted in downregulation of transcript levels of five lincRNAs that function to repress lineage differentiation programme (Fig. 5d).

Next, we examined whether lincRNA transcription is coregulated by Oct4 and Pontin as in the case of protein-coding gene transcription. Thus, we designed luciferase reporter constructs of the Oct4-dependent *linc1253* promoter and examined whether Oct4-binding site is crucial for the activity of *linc1253* promoter. We constructed two different *linc1253* promoter-luciferase reporters, one containing Oct4-binding region (*linc1253*-*1000*) and the other with deleted Oct4-binding region (*linc1253*-*500*). Introduction of Pontin further increased Oct4-dependent *linc1253*-*1000* promoter-luciferase activity (Fig. 5e). Introduction of neither Oct4 nor Pontin increased *linc1253*-*500* promoter-luciferase activity. Consistently, Oct4-dependent induction of luciferase activity from *linc1253*-*1000* promoter was attenuated by knockdown of Pontin (Fig. 5e), suggesting that Pontin functions as a coactivator of Oct4-dependent lincRNA transcription. Together, these results indicate that coactivator function of Pontin is mediated by Oct4 binding to the *linc1253* promoter.

### Characterization of *linc1253* that is cooperatively regulated by Oct4-Pontin module

**Figure 6:**
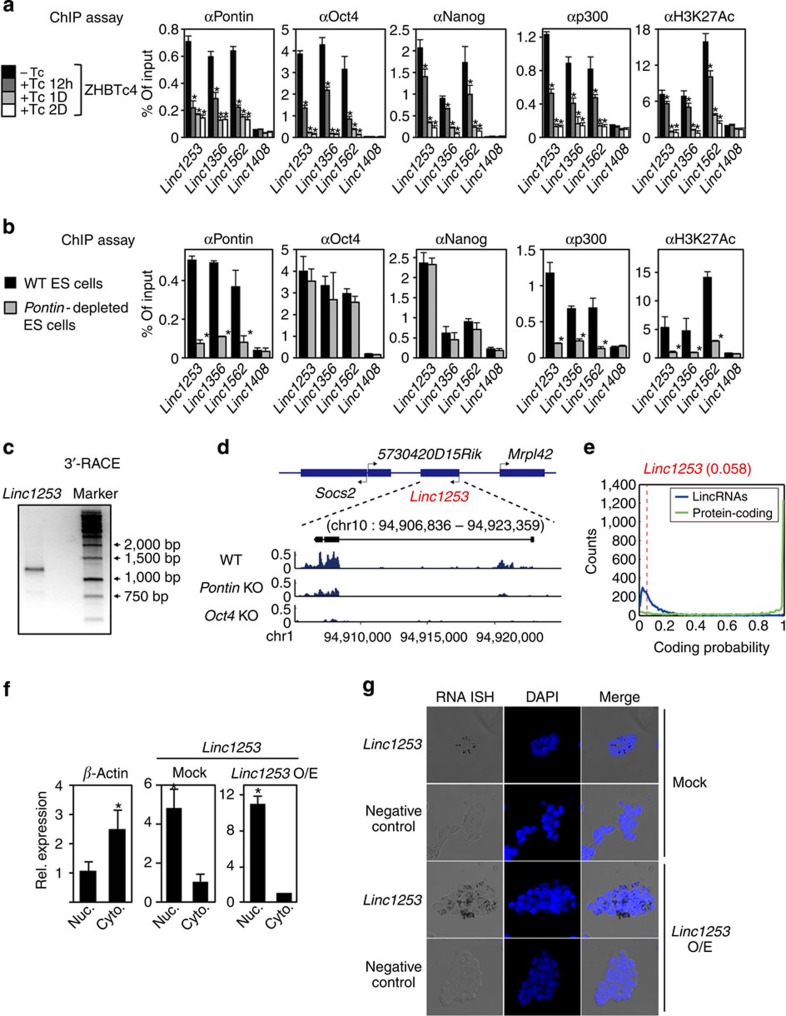
Characterization of *linc1253*, a Pontin-dependent Oct4 target lincRNA. (**a**,**b**) ChIP assays were performed on Pontin-dependent Oct4 target lincRNA loci with indicated antibodies in ZHBTc4 ES cells after vehicle or tetracycline treatment for indicated times (**a**) or in WT and *Pontin*-depleted ES cells (**b**). *Linc1408* is a negative control locus. Values are expressed as mean±s.d. of three independent experiments. *P<0.05. (**c**) 3′-RACE analysis of *linc1253* in ES cells. (**d**) Transcript structure and mRNA-sequencing reads of *linc1253* genes in WT, *Pontin*-depleted and *Oct4*-depleted ES cells. *Y* axis represents the read coverage for each base in *linc1253*. (**e**) Coding probability distributions of protein-coding genes (green) and lincRNAs (blue). A low coding probability of *linc1253* (0.058, red dotted line) suggests that *linc1253* is non-coding. (**f**) ZHBTc4 ES cells were fractionated into nuclear and cytoplasmic extracts. *β-Actin* and *linc1253* transcripts were quantified in both fractions by quantitative RT-PCR. Values are expressed as mean±s.d. of three independent experiments. *P<0.05. (**g**) Analysis of *linc1253* transcripts in ES cells by chromogenic RNA *in situ* hybridization. Antisense probes for *linc1253* or negative control probes were hybridized to fixed ES cells infected with Mock or *linc1253* overexpression (*linc1253* O/E) lentiviruses. *linc1253* expression was shown by chromogenic signals (dark dots) and nuclei were stained with DAPI (blue). Representative images are shown. Similar results were obtained from three independent experiments. Magnification × 40.

To further investigate whether Pontin is recruited to lincRNA loci through Oct4 binding sites along with Oct4 and p300 in ES cells, we performed ChIP assays on the *linc1253*, *linc1356* and *linc1562* loci in WT, *Oct4*-depleted and *Pontin*-depleted ES cells. For these lincRNA loci, Pontin and Oct4, along with p300, were corecruited to Oct4-binding sites, concomitant with strong H3K27 acetylation signal in WT ES cells (Fig. 6a). Similar to other Oct4-Pontin target genes, depletion of Oct4 led to the failure of recruitment of Pontin, Nanog and p300, with reduction of H3K27 acetylation levels (Fig. 6a). The recruitment of Oct4 and Nanog to the *linc1253*, *linc1356* and *linc1562* loci were not affected by lack of Pontin, but the recruitment of p300 along with H3K27 acetylation levels was drastically decreased in *Pontin*-depleted ES cells (Fig. 6b). These data confirm that Pontin functions as a transcriptional coactivator along with p300 for Oct4-Pontin target lincRNAs in an Oct4-dependent manner. Among the Oct4-Pontin target lincRNAs, *linc1253* is one of the most significantly downregulated lincRNAs in both *Pontin*-depleted ES cells and *Oct4*-depleted ES cells (Supplementary Fig. 4a and Supplementary Data 4).

We further characterized the gene/isoform structure, genomic location, coding probability and subcellular localization of *linc1253*. The mRNA-sequencing data of human ES cells (H1 and H9 cells)^45^ revealed no evidence for *linc1253* expression, suggesting that *linc1253* may not be conserved in humans. On the basis of the 3′ rapid amplification of cDNA ends (RACE) experiment followed by cDNA cloning, we found that *linc1253* is an ∼1,500-nucleotide transcript comprising three exons at the locus of chr10: 94,906,836–94,923,359 (minus strand) (Fig. 6c), which is consistent with our mRNA-sequencing data in ES cells (Fig. 6d). According to the UCSC genome browser, there were two mouse expressed sequence tags (CJ063160 and CJ133752) covering this locus. Results from the 3′ RACE and mRNA sequencing analyses indicate that there are no isoforms at least in ES cells. Next, we assessed coding potential across the mature RNAs using the Coding Potential Assessment Tool (CPAT)^46^. According to the coding probability distribution of protein-coding genes and lincRNAs, *linc1253* showed a very low coding probability of 0.058, indicating that this transcript is non-coding (Fig. 6e). We quantified the *linc1253* transcript levels in both nuclear and̀ cytoplasmic fractions of ES cells by quantitative RT-PCR analysis, and found that *linc1253* mainly resides in the nucleus (Fig. 6f). *In situ* hybridization analysis of *linc1253* confirmed exclusive localization of *linc1253* in the nucleus (Fig. 6g).

### Molecular mechanism of regulation of *linc1253* by the Oct4-Pontin module

**Figure 7:**
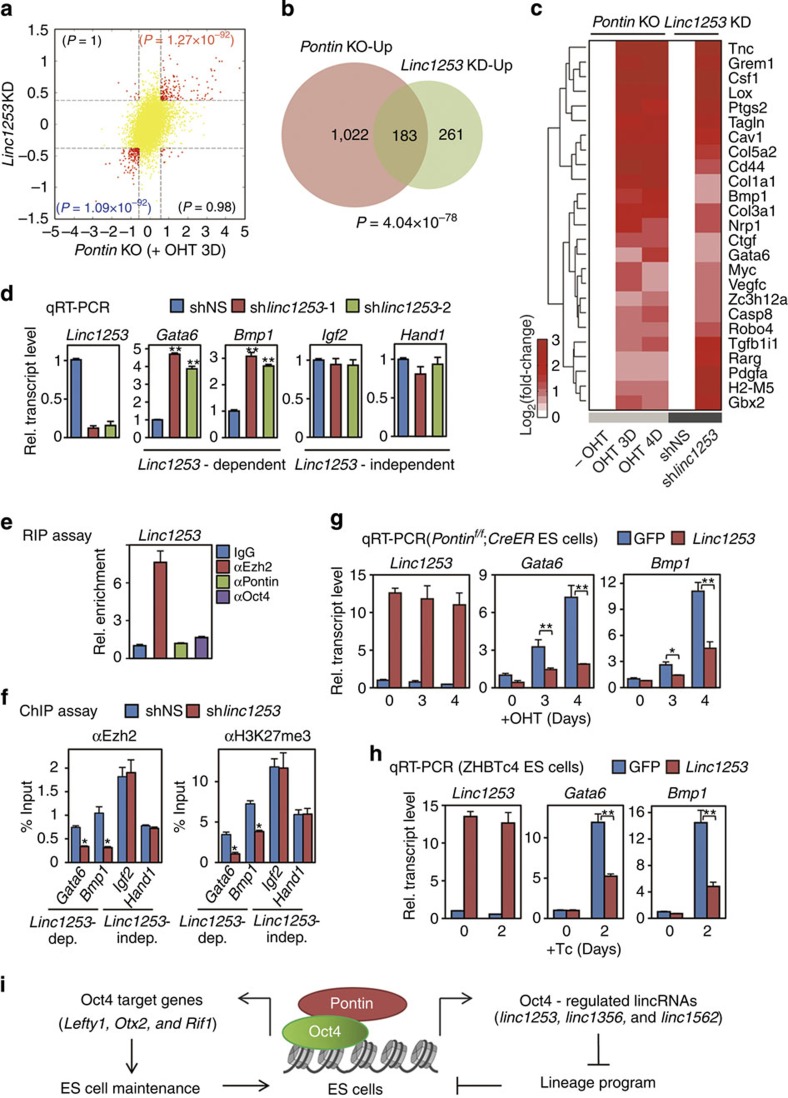
Molecular mechanism of cooperative regulation of *linc1253* by Oct4-Pontin module. (**a**) Comparison of log_2_-fold-changes between *Pontin*-depleted (OHT 3D) and *linc1253* knockdown ES cells. The scatter plot shows the DEGs (red dots) with consistent directions in *Pontin*-depleted ES cells and *linc1253* knockdown ES cells and non-DEGs (yellow dots). The significance for the number of genes in each quadrant was computed by Fisher's exact test. (**b**) Venn diagram representing the relationship between the upregulated genes in *Pontin-*depleted (Pontin KO-Up) ES cells and *linc1253* knockdown (linc1253 KD-Up) ES cells. (**c**) The genes related to differentiation among commonly upregulated by depletion of *Pontin* (OHT 3D and 4D) and knockdown of *linc1253* in ES cells. Colour bar represents the gradient of normalized log_2_-fold-changes. (**d**) Transcript levels of genes related to lineage specification or differentiation induced by *linc1253* knockdown. For knockdown of *linc1253*, lentiviral shRNA against *linc1253* was infected into ZHBTc4 ES cells. The quantity of lincRNA and mRNA was normalized by using primers to detect *Gapdh*. Values are expressed as mean±s.d. of three independent experiments. **P<0.01. NS=not statistically significant. (**e**) RNA-IP (RIP) assay. RIP of *linc1253* was performed using anti-Ezh2, anti-Pontin or anti-Oct4 antibodies. RIP enrichment was measured by qRT-PCR and values were normalized to background immuno-precipitation measured by IgG. Values are expressed as mean±s.d. of three independent experiments. (**f**) ChIP assays were performed on indicated gene loci for Ezh2 and trimethylation of histone H3K27 in ES cells infected with shNS (blue bars) or sh*linc1253* (red bars) lentiviruses. Values are expressed as mean±s.d. of three independent experiments. *P<0.05. (**g**,**h**) Effects of ectopic expression of linc1253 on *Gata6* and *Bmp1* genes involved in differentiation in *Pontin*-depleted ES cells (**g**) and *Oct4-*depleted ES cells (**h**). Values are expressed as mean±s.d. of three independent experiments. **P<0.01. (**i**) Schematics showing function of Pontin as a transcriptional coactivator for Oct4 target protein-coding genes involved in the ES cell maintenance and Oct4 target lincRNAs involved in repression of differentiation processes in ES cells.

To compare the genes regulated by *linc1253* and Pontin, we further performed mRNA sequencing of ES cells after knockdown of *linc1253* by lentiviral shRNA and identified 838 DEGs (Supplementary Data 1,3). Consistent with the DEGs in *Pontin*-depleted ES cells, the upregulated genes in *linc1253* knockdown ES cells are mainly involved in differentiation and development, whereas the downregulated genes are involved in metabolism and ion homeostasis (Supplementary Data 2). The log_2_-fold-changes in *linc1253*-knockdown ES cells showed a high positive correlation with those in *Pontin*-depleted ES cells (Fig. 7a and Supplementary Fig. 4b). Consistently, the upregulated genes by *linc1253* knockdown significantly (*P*=4.04 × 10^−78^ by Fisher's exact test) overlapped with those by *Pontin* depletion (Fig. 7b). Among the commonly upregulated genes by the depletions of *linc1253* and Pontin in ES cells (Fig. 7c), we selected two representative development-related genes (*Gata6* and *Bmp1*). Quantitative RT-PCR analysis confirmed that loss of *linc1253* increased transcript levels of *Gata6* and *Bmp1*, although those of *Igf2* and *Hand1* were not changed (Fig. 7d). These results indicate that *linc1253* regulated by the Oct4-Pontin module is involved in the repression of a subset of developmental genes in ES cells.

Ezh2 is a key component of polycomb repressive complex (PRC) 2 and possesses a methyltransferase activity on histone H3K27. Recent studies have reported that nuclear lincRNAs are functionally linked to chromatin-modifying proteins containing Ezh2 and various transcription factors^47^,^48^. To examine whether *linc1253* has a role in Ezh2-mediated repression of developmental genes, we tested the possibility that *linc1253* physically associates with Ezh2. We performed RNA immunoprecipitation (RIP) assays using antibodies specific to Ezh2, Pontin and Oct4, and found that Ezh2 associates with *linc1253* (Fig. 7e). Neither Pontin nor Oct4 exhibited comparable binding to *linc1253* (Fig. 7e). We then performed ChIP assays in ES cells after knockdown of *linc1253* by lentiviral shRNA to determine whether Ezh2 recruitment and tri-methylation of histone H3K27 on target gene loci are affected by loss of *linc1253*. On depletion of *linc1253*, recruitment of Ezh2 concomitant with H3K27 tri-methylation was significantly diminished to the *linc1253*-dependent target gene loci (Fig. 7f). These results indicate that *linc1253* collaborates with Ezh2 to exert its repressive functions on target genes.

In addition to loss-of-function studies, we carried out gain-of-function studies for *linc1253*. We examined the effect of *linc1253* overexpression in reprogramming efficiency and found that *linc1253* overexpression did not affect reprogramming efficiency (Supplementary Fig. 4c). To determine whether overexpression of *linc1253* reverses upregulation of developmental genes in *Pontin*-depleted ES cells and *Oct4*-depleted ES cells, we transduced *Pontin*^*f/f*^; *CreER* ES cells or ZHBTc4 ES cells with lentivirus expressing control GFP or *linc1253*. In GFP-infected ES cells, depletion of either Pontin or Oct4 induced expression of *Gata6* and *Bmp1* drastically (Fig. 7g,h). However, drastic induction of *Gata6* and *Bmp1* expression in *Pontin*-depleted and *Oct4*-depleted ES cells was significantly attenuated by overexpression of *linc1253* (Fig. 7g,h). These data indicate that ectopic expression of *linc1253*, at least partially, attenuated upregulation of developmental genes in *Pontin*-depleted ES cells and *Oct4*-depleted ES cells. Our findings demonstrate that *linc1253* functions as an important factor acting in the downstream of the Oct4-Pontin axis, attenuating a subset of genes involved in lineage programme in ES cells.

## Discussion

Our findings demonstrate a regulatory network in ES cells whereby Pontin directly activates the expression of both lincRNAs involved in repression of differentiation processes and protein-coding genes required for the ES maintenance as a transcriptional coactivator in an Oct4-dependent manner (Fig. 7i). It has been shown that lincRNAs conduct diverse and distinct biological functions including *trans*-acting gene regulation (HOTAIR)^49^, imprinting (Air and H19)^50^,^51^, X-chromosome inactivation (Xist and Tsix)^52^, nuclear shuttling (Nron)^53^ and somatic tissue differentiation (*Braveheart* and TINCR)^54^,^55^. Although lincRNAs identified in ES cells have been shown to regulate the cell states by both maintaining the pluripotency programme and repressing differentiation programme, it remains largely unknown how these lincRNAs are controlled and which factors lie upstream.

We identified Pontin as a key factor for Oct4-dependent lincRNA transcription processes in ES cells. Pontin and Oct4 show functional cooperation for the transcriptional activation of Oct4 target lincRNAs. It is intriguing that the mode of Oct4 and Pontin for the regulation of lincRNA transcription is similar to that of protein-coding gene transcription. Pontin activates Oct4-dependent lincRNA transcription as a coactivator and the binding of Pontin to the lincRNA loci is mediated by Oct4. Although the functional outcome of transcriptional activation of Oct4 target lincRNAs and Oct4-dependent protein-coding genes by Pontin is distinct, the net result is to favour ES cell maintenance. Pontin appears to acquire maximum efficiency to maintain ES cell identity by regulating transcription process of both lincRNAs and protein-coding genes in an Oct4-dependent manner. It could be one of the prototypes for chromatin modifiers to regulate ES cell identity. Our finding that a group of Oct4 target genes are not dependent on Pontin suggests that Pontin recruitment to Oct4 binding sites may be context-dependent and/or regulatory element-dependent. It will be challenging to find what could be determinants to decide Pontin dependency to the Oct4 target genes.

Intriguingly, Oct4 binding to target genes is not affected by Pontin, but lack of Pontin is sufficient for perturbation of ES cell maintenance. On the basis of the failure of p300 recruitment to the Oct4-Pontin target genes in *Pontin*-depleted ES cells, we speculate that Pontin accommodates p300 recruitment for transcriptional activation of Oct4-Pontin target genes. Therefore, Pontin functions as a critical factor for p300 recruitment to Oct4-Pontin target genes. It is consistent with the notion that chromatin remodelling process is preceded by histone modifications to initiate efficient transcription processes. Some histone-modifying enzymes have been shown to have a variety of crucial functions for ES cell maintenance. A histone methyltransferase Ezh2 is required for the early embryogenesis and establishment of ES cell^56^. Ring1A/B, the core components of PRC1, are required for the maintenance of ES cell identity. In the *Ring1A/B*-double KO ES cells, proliferation is halted and the cells lose typical ES cell morphology^57^. Depletion of *Mll2* histone methyltransferase causes increase of apoptosis and skewed differentiation with decreased SSEA-1 expression in ES cells^58^.

Together, our findings demonstrate that Pontin, Oct4 and lincRNAs are important regulatory components within the ES cell circuitry and efficiently orchestrate the cell fate programme by forming a functional module. We anticipate that identification of new ES cell-specific lincRNAs associated with chromatin modifiers will lead to a better understanding of cell fate decision programme.

## Methods

### Generation of conditional *Pontin*-deficient mice

To create a conditional targeting vector in which exon 3 of the *Pontin* gene was flanked by *loxP* sites, a 13-kb region used to construct the targeting vector was first subcloned from a BAC clone (bMQ403n16, Source BioScience) into a pBluescript phagemid system. The *FRT*-flanked puromycin cassette containing a *loxP* sequence was inserted at the 3′ and the single *loxP* site was inserted at the 5′ of exon 3. The target region was ∼2.5 kb and included exon 3. Twenty micrograms of the targeting vector was linearized by NotI restriction enzyme and then transfected to E14Tg2A ES cells by electroporation. After puromycin selection, surviving clones were expanded to identify recombinant ES clones by Southern blot analysis. For XbaI digestion, the bands representing WT and mutant alleles are 13 and 5.2 kb, respectively. Targeted ES cells were microinjected into C57BL/6 blastocysts that were used to generate chimeras. The male chimeras were mated to C57BL/6 female mice to obtain F1 heterozygous offspring. Puromycin selection cassette was deleted by crossing targeted heterozygous F1 with Flp deleter strain (FLPeR mice, The Jackson Laboratory strain 003946). All mice used for this work were backcrossed to C57BL/6 at least five generations. This study was reviewed and approved by the Institutional Animal Care and Use Committee (IACUC) of National Cancer Center Research Institute and Seoul National University.

### Genotyping

The primers used in PCR analysis for genotyping heterozygous mice are: Pair A 5′-ACTCACTCTGTGGAGCAGAC-3′ and 5′-ACCTTACCTGCGCTCCCATC-3′; Pair B 5′-CTACAGTCTCAGCACTCAGG-3′ and 5′-CCATTTGTCACGTCCTGCAC-3′; Pair C 5′-CAACCTCCCCTTCTACGAGC-3′ and 5′-ACCTTACCTGCGCTCCCATC-3′. The primers used in PCR analysis for genotyping floxed alleles and deleted alleles are: 5′-TCGAGGCAGGAGTACCAGGC-3′, 5′-TTCAGGACAGCAGACTCTGG-3′ and 5′-CTCTGCCTGTGAAACCATACC-3′.

### Antibodies

The following commercially available antibodies were used: anti-Oct4 (C-10) and anti-p300 (C-20, N-15, and H-272) antibodies from Santa Cruz Biotechnology; anti-Pontin (SAB4200194) antibody from Sigma-Aldrich; anti-Nanog (ab21624) and anti-H3K27Ac (ab4729) antibodies from Abcam; anti-H3 (#2650) antibody from Cell Signaling Technology; anti-Ezh2 (#612667) antibody from BD bioscience; and anti-SSEA-1 (MAB2155) antibody from R&D systems. Working dilutions or quantities of the antibodies used in the study are summarized in Supplementary Table 1.

### Quantitative real-time RT-PCR

The abundance of mRNA and lincRNA was detected by an ABI prism 7300 system with SYBR Green (Enzynomics). The quantity of mRNA and lincRNA was calculated using DDCt method and normalized by using primers to detect *Gapdh*. All reactions were performed as triplicates. The following primers were used: *Oct4* forward 5′-GGCTTCAGACTTCGCCTCC-3′, reverse 5′-AACCTGAGGTCCACAGTATGC-3′; *Nanog* forward 5′-TCTTCCTGGTCCCCACAGTTT-3′, reverse 5′-GCAAGAATAGTTCTCGGGATGAA-3′; *Ptch1* forward 5′-AAAGAACTGCGGCAAGTTTTTG-3′, reverse 5′-CTTCTCCTATCTTCTGACGGGT-3′; *Rif1* forward 5′-ACTGACTCCGGGACATAAAGG-3′, reverse 5′-ATAGAAGGGATTGCAGCCATTC-3′; *Tpm1* forward 5′-CAGAAGGCAAATGTGCCG AG-3′, reverse 5′-TCCAGCATCTGGTGC ATACTA-3′; *Timp2* forward 5′-TCAGAGCCAAAGCAGTGAGC-3′, reverse 5′-GCCGTG TAGATAAACTCGATGTC-3′; *Lefty1* forward 5′-CCAACCGCACTGCCCTTAT-3′, reverse 5′-CGCGAAACGAACCAACTTGT-3′; *Otx2* forward 5′-TATCTAAAGCAACCGCCTTAC G-3′, reverse 5′-AAGTCCATACCCGAAGTGGTC-3′; *Fut9* forward 5′-TCGCCCATTTCTAATCGTCTGC-3′, reverse 5′-AGACTCCATTGGACTGAAGACC-3′; *Gata6* forward 5′-TTGCTCCGGTAACAGCAGTG-3′, reverse 5′-GTGGTCGCTTGTGTAGAAGGA-3′; *Igf2* forward 5′-GTGCTGCATCGCTGCTTAC-3′, reverse 5′-GACAAACTGAAGCGTGTCAA C-3′; *Krt18* forward 5′-CAGCCAGCGTCTATGCAGG-3′, reverse 5′-CTTTCTCGGTCTG GATTCCAC-3′; *Mycn* forward 5′-ACCATGCCGGGGATGATCT-3′, reverse 5′-ATCTCCG TAGCCCAATTCGAG-3′; *Bmp1* forward 5′- TTGTACGCGAGAACATACAGC-3′, reverse 5′-CTGAGTCGGGTCCTT TGGC-3′; *Hand1* forward 5′-GGCAGCTACGCACA TCATCA-3′, reverse 5′-CCTGGCATCGGGACCATAG-3′; *linc1253* forward 5′-TGCAGGTTCATAATTCATGGC-3′, reverse 5′-AATGGAATGCTTTGTCACCAC-3′; *linc1562* forward 5′-CTG GATCTGAGAGACGACCC-3′, reverse 5′-GAAATGCTCTGGAGACGGAG-3′; *linc1356* forward 5′-TCTGTTTCCGAATTGAAGGC-3′, reverse 5′-GTTTCCCAAATCAGCAGCTC-3′; *linc1517* forward 5′-TTATACCGAAACCGGGAACTC-3′, reverse 5′-AGCAAAGCTGGTCAGGAGAC-3′; *linc1602* forward 5′-CCTGAGCCTTCTGTGGTCTC-3′, reverse 5′-CTCTTGGAGTGCTTCATCTGG-3′. Values are expressed as mean±s.d. of three independent experiments.

### GST pull-down assays

The recombinant GST-Pontin was expressed using the pGEX-4T1-Pontin vectors. The coding regions of Oct4 were inserted into the pcDNA vector containing T7 promoter. ^35^S-methione-labelled Oct4 was produced using an *in vitro* transcription and translation assay kit (TNT Quick Coupled Transcription/Translation system; Promega), according to the manufacturer's instructions.

### ChIP and two-step ChIP assays

The ChIP assays were conducted as described. Cells were crosslinked with 1% formaldehyde for 10 min at room temperature, and formaldehyde was inactivated by the addition of 125 mM glycine. Chromatin extracts containing DNA fragments with an average size of 400 bp were immmunoprecipitated by using antibodies. Quantitative PCR was used to measure enrichment of bound DNA, and the value of enrichment was calculated by relative amount to input and ratio to IgG. All reactions were performed in triplicates. For the two-step ChIP assays, components were eluted from the first immunoprecipitation reaction by incubation with 10 mM dithiothreitol at 37 °C for 30 min and diluted 1:50 in ChIP dilution buffer (20 mM Tris-HCl, pH 8.1, 150 mM NaCl, 2 mM EDTA and 1% Triton X-100) followed by re-immunoprecipitation with the second antibodies. Two-step ChIP assay was performed in essentially the same way as the first immunoprecipitations. The following primers were used: *Lefty1* forward 5′-CTGGATTGTCTTTGGGGAAA-3′, reverse 5′-CCCCAATCCACATTCA CTTC-3′; *Otx2* forward 5′-CTCCAAATGCACGCTCTACA-3′, reverse 5′-TAGCTAGTGC CAGCCAATGA-3′; *Rif1* forward 5′-GTCCCCACTCTCAGAAGCTG-3′, reverse 5′-ACGC ATTCAAGCTTTGGTCT-3′; *Mycn* forward 5′-TTAGCGAATCCTTGCTACCG-3′, reverse 5′-CTTCGGAAAGGCTTTTGTTG-3′; *linc1253* forward 5′-TTGCCTCCTCAAGAAATGC T-3′, reverse 5′-CATGGCTCCAGTTCCTCTGT-3′; *linc1356* forward 5′-ACCCA CAGGCT CCTAGGTTT-3′, reverse 5′-TCCAAGCTGTTCTCCCAACT-3′; *linc1562* forward 5′-CAGAGAGAGGGAAGCAATGG-3′, reverse 5′-GAATGGCTCAGTGTGGGAAT-3′. Values are expressed as mean±s.d. of three independent experiments.

### Construction of reporter plasmids and luciferase reporter assay

Each promoter region was cloned into a pGL2-basic reporter vector (Promega) by using the PCR method. Oct4-binding sites were identified from previous reports^18^,^41^,^59^. The primers used were: *linc1253*-1000 XhoI forward 5′-CACCTCGAGTGGAGCTTCAGTCCCCGA-3′, KpnI reverse 5′-CACGGTACCGTAAGATGGGAATATTGTCTGG-3′; *linc1253*-500 XhoI forward 5′-CACCTCGAGTGGAGCTTCAGTCCCCGA-3′, KpnI reverse 5′-CACGGTACC TTATGTGCTAGTTAGGGTAACT-3′; *Rif1*-800 KpnI forward 5′-CACGGTACCTGTGGA GAGTGCTGAGAGG-3′, XhoI reverse 5′-CACCTCGAGCCTGACTCCAGCTACTTGC-3′; *Rif1*-640 KpnI forward 5′-CACGGTACCCATGGGTCTCCTTTAGCAAC-3′, XhoI reverse 5′-CACCTCGAGCCTGACTCCAGCTACTTGC-3′. About 0.2–0.4 μg of pCAG-Oct4, 0.3–0.6 μg of pCAG-Pontin, 0.2 μg of pGL2-*Rif1* promoter-luciferase and 0.1 μg of pGL2-*linc1253* promoter-luciferase plasmid were used for co-transfection. For knockdown of Pontin, 10–20 μl of pLKO-shPontin lentiviral stock was used. 293 T cells were transiently transfected with each promoter-luciferase reporter plasmid using PEI transfection reagent (Sigma-Aldrich). Luciferase activity was measured in a luminometer at 48 h after transfection and normalized by β-galactosidase expression with a Luciferase system (Promega). Values are expressed as means±s.d. of three independent experiments.

### Lentivirus construction and production

To knockdown *Pontin, p300* or *linc1253*, the shRNA pLKO.1 lentiviral vectors targeting Pontin (pLKO-shPontin), p300 (pLKO-shp300) or linc1253 (pLKO-shlinc1253) were cloned from targeting sequences. The targeting hairpin sequences are linc1253 5′-GTGTAGGAGCTGGGATGAAAT-3′, p300 5′- AATACCTCGTGATGCCACTTA-3′ and Pontin 5′-AAGGGGAGGTGACAGAGCTCA-3′. For ectopic expression of *linc1253*, we cloned *linc1253* cDNA into the pLJM1 lentivirus vector (pLJM1-linc1253). On the basis of the size determined by 3′RACE PCR, *linc1253* cDNA was PCR-amplified using the following primers: forward 5′-CCAACCGGTGGAAATGGAATGCTTTGTCAC-3′, reverse 5′- CCAGAATTCCCCTGGGACTAATGGAGGT-3′. For ectopic expression of Pontin, we cloned *Pontin* cDNA into the same vector. Lentivirus production was performed as described. The lentiviral vector was co-transfected with packaging vectors (psPAX2 and VSV-G) into 293 T cells. The resultant supernatant was collected at 48 h after transfection and filtered through a 0.45-μm membrane. For concentration of lentivirus, Retro-X concentrator was used according to the manufacturer's instructions (Clonetech). At 48 h after transduction, puromycin (1 μg ml^−1^) was added to the medium to select transduced cells.

### FACS analysis

Cell cycle profiling of propidium iodide stained cells was performed as described. Fractions of cells in each phase were quantified using FlowJo software. Cells were trypsinized and then fixed in 70% ethanol at 4 °C. After fixation, the cells were incubated with RNase A (10 μg ml^−1^), Nonidet P-40 (0.05%) and propidium iodide (50 μg ml^−1^) for 1 h. For the BrdU FACS analysis, cells were incubated for 10 min in the presence of BrdU (10 μM). Harvested cells were fixed in 70% ethanol at 4 °C and denatured in 2 N HCl, 0.5% Triton X-100 for 1 h. The cells were then neutralized with 0.1 M Na_2_B_4_O_7_ (pH 8.5) and incubated with a BrdU antibody and FITC-conjugated secondary antibody in PBS containing 1% BSA and 0.5% Tween 20 for 1 h each. Cells were stained with propidium iodide solution and then analysed using the FACS Caliber flow cytometer (BD Biosciences). Cell cycle and DNA contents were analysed using FlowJo software.

### ES cell culture

Mouse ES cells were cultured as described previously^59^. In brief, ES cells were either co-cultured with mouse primary embryonic fibroblast feeders or cultured under feeder-free conditions. ES cells were maintained in Dulbecco's modified Eagle medium (DMEM; Welgene), supplemented with 15% fetal bovine serum (FBS; Hyclone), 0.055 mM β-mercaptoethanol, 2 mM L-glutamine, 0.1 mM nonessential amino acid, 5,000 units ml^−1^ of penicillin/streptomycin (GIBCO) and 1,000 units ml^−1^ of leukaemia inhibitory factor (LIF) (Chemicon). A conditional *Oct4*-depleted (ZHBTc4) ES cell line was described previously^42^. *Oct4*-depleted cells were generated by treating ZHBTc4 ES cells with tetracycline for 2 days. *Pontin*^*f/f*^; *CreER* ES cells were derived from blastocysts from intercrosses between *Pontin*^*f/f*^; *CreER* and *Pontin*^*f/f*^ mice. *CreER* transgenic mice were purchased from Jackson laboratory. *Pontin*-depleted ES cells were generated by treating *Pontin*^*f/f*^; *CreER* ES cells with OHT for 3 or 4 days. Immunoblot analyses showing depletion of *Pontin* or *Oct4* were independently repeated three times.

### Co-immunoprecipitation

ZHBTc4 ES cells were cultured and lysed with lysis buffer (200 mM NaCl, 50 mM Tris-HCl, pH 8.0 and 0.5% NP40). About 20 mg of ES cell extracts was immunoprecipitated with each 2 μg of control IgG, anti-Oct4 antibody, anti-Nanog antibody or anti-Pontin antibody overnight and then incubated with 50 μl (50% slurry) of protein A agarose beads for 1 h. The immunoprecipitated materials were washed with 500 μl of washing buffer (150 mM NaCl, 50 mM Tris-HCl, pH 8.0, and 0.5% NP40) for four times and bound materials were eluted by boiling in 50 μl of sampling buffer (2% 2-mercaptoethanol, 5% glycerol, 1% SDS and 60 mM Tris-HCl, pH 6.8) and subjected to immunoblot analysis. Protein samples were resolved with 12% SDS polyacrylamide gel, and 25 μl of eluted samples were loaded in each well. The resolved proteins were transferred to PVDF membranes, and the membranes were incubated with primary antibodies (1/1,000) at 4 °C overnight, washed and detected using HRP-conjugated secondary antibodies (light chain-specific, Jackson laboratory). Images of the immunoblots were visualized and recorded using the LAS 4000-mini system (Fujifilm). These experiments were independently repeated three times.

### 3′- RACE

To determine the size of *linc1253*, we performed 3′-RACE as previously described^60^. In brief, RACE was carried out by using 1.5 μg of total RNA extracted from ZHBTc4 ES cells. The Oligo-dT adapter primer (5′-GCTCGCGAGCGCGTTTAAACGCGCACGCGTTTTTTTTT TTTTTTTTT-3′) was used in the reverse transcription. The first PCR reaction was performed by using *linc1253* forward 1 primer (5′-AATGGAATGCTTTGTCACCAC-3′) and the first adaptor primer (5′-GCTCGCGAGCGCGTTTAAAC-3′). Amplification was performed as follows: initial denaturation for 1 min at 95 °C, denaturation for 30 s at 95 °C, annealing for 30 s at 60 °C, extension for 5 min at 72 °C and repeated for 35 cycles. The obtained band was gel purified and PCR-amplified with *linc1253* forward 2 primer (5′-CCACTCCTCCGA GCAACACAG-3′) and the second adaptor primer (5′-GCGTTTAAACGCGCACGCGT-3′). Amplification was performed as follows: initial denaturation for 1 min at 95 °C, denaturation for 30 s at 95 °C, annealing for 30 s at 56 °C, extension for 1 min at 72 °C and repeated for 35 cycles. The obtained band was gel purified and sequenced. These experiments were independently repeated three times.

### mRNA and ChIP sequencing

We obtained total RNAs from (1) *Pontin*^*f/f*^*; CreER* ES cells at 0, 3 or 4 days post treatment with OHT for *Pontin*-depleted ES cells, (2) ZHBTc4 ES cells at 2 days post treatment with tetracycline for *Oct4*-depleted ES cells and (3) ZHBTc4 ES cells infected by pLKO-shLuciferase (shNS) or pLKO-shlinc1253 lentivirus at 4 days post infection for knockdown of linc1253. Poly(A) mRNA isolation from total RNA (5 μg) and fragmentation was performed using the Illumina Truseq RNA Sample Prep Kit with poly-T oligo-attached magnetic beads, according to the manufacturer's instructions. Reverse transcription of RNA fragments was performed using Superscript II reverse transcriptase (Life Technologies). The adaptor-ligated library was size-selected by band excision after agarose gel electrophoresis and purified using the QIAquick gel extraction kit (Qiagen). The prepared libraries were sequenced on an Illumina Hi-Seq 2000 (DNA Link, Korea) and Hi-Seq 2500 (NICEM, Seoul National University). A ChIP assay for preparation of ChIP-seq libraries was carried out as described previously^59^. In brief, ZHBTc4 ES cells were crosslinked with 1% formaldehyde for 10 min at room temperature, and formaldehyde was inactivated by the addition of 125 mM glycine. Chromatin extracts containing DNA fragments with an average size of 400 bp were immmunoprecipitated by using antibodies against GFP (control) or Pontin. Eluted ChIP DNA was used for preparing the libraries using the NEXTflex ChIP-seq kit (Illumina), according to the manufacturer's instructions. The prepared libraries were sequenced on an Illumina Hi-seq 2500 (NICEM, Seoul National University).

### Analysis of mRNA- and ChIP-sequencing data

For both sequencing data, we first removed adapter sequences (TrueSeq universal and index adapters) and then trimmed the ends of the adapter-free reads for which PHRED scores were lower than 20 using the cutadapter software^61^. Remaining reads were then aligned to the mouse reference genome (NCBIM 37) using TopHat aligner^62^ for mRNA sequencing and using Bowtie2 (ref. 63) for ChIP sequencing. For the mRNA-sequencing data, considering the variations in individual genomes and presence of multiple gene copies, we used two mismatches in a read and allowed the reads to be aligned in up to 10 different locations, which are default options in the TopHat aligner. After the alignment, we counted the number of reads mapped to gene features (GTF file of NCBIM 37) using HTSeq. To reduce the technical variations across the samples, we normalized the read counts using the TMM method^64^ that uses RNA compositions and library sizes between the samples provided by edgeR package^65^ in *R*. For the ChIP-sequencing data, among the aligned reads, only the reads uniquely aligned and with MAPQ scores >5 were used for further analysis.

### Identification of DEGs

We first identified ‘expressed' genes as the genes with normalized read counts (counts per million) >1 under at least one of the four conditions (WT ES cell, *Pontin*-depleted ES cells (3 days and 4 days post-OHT treatment) and *Oct4*-depleted ES cells (2 days post-Tc treatment)). For these expressed genes, we computed log_2_-read counts after adding one to the normalized read counts and then log_2_-fold-changes in the comparison of *Pontin*^*f/f*^; *CreER* ES cells at 3 days or 4 days post-OHT treatment (*Pontin*-depleted ES cells) and ZHBTc4 ES cells at 2 days post-tetracyclin treatment (*Oct4*-depleted ES cells) versus *Pontin*^*f/f*^; *CreER* ES cells at 0 days post-OHT treatment (WT ES cells). Using the log_2_-fold-changes for each comparison, we identified the genes as DEGs with absolute log_2_-fold changes >0.58 (1.5-fold). To identify DEGs by *linc1253* knockdown, we first selected ‘expressed' genes with normalized read counts >1 under at least one of the four samples, duplicated samples of each WT ES cells and *linc1253*-knockdown ES cells. From these expressed genes, we performed the exact test in edgeR package and log_2_-median ratio test. After separately computing *P* values from the two tests, we combined the *P* values using Stouffer's method^66^ and then selected the DEGs by *linc1253* knockdown as the ones with the combined *P*<0.05. False positives were further reduced by excluding the genes with log_2_-median ratio<0.378, the mean of 2.5th and 97.5th percentiles of the log_2_-median ratios obtained from random permutations of the four samples.

### Functional enrichment analysis

To identify cellular processes represented by a set of DEGs (for example, up- and downregulated genes by *Pontin* depletion, *Oct4* depletion or *linc1253* knockdown), we performed functional enrichment analysis for the DEGs using DAVID Bioinformatics Resources V6.7 (ref. 40 and selected the Gene Ontology Biological Process (GOBP) with *P* value <0.1 and gene count ≥3 as the ones represented by the DEGs.

### Comparisons of ES cell gene expression profiles

Previously reported gene expression profiles of *Oct4*, *Nanog*, *Tip60*, *p400* or *Dnmt1*-depleted ES cells^10^,^57^,^59^ were used to evaluate the similarity in the effects of depletion of these factors in ES cells. For Agilent microarray data (GSE11243), we normalized probe intensities using the quantile normalization method. For Affymetrix microarray data (GSE4189 and GSE10519), we normalized probeset intensities using the GC-RMA method^67^. Using the normalized data, we computed log_2_-fold-changes in ES cells deficient of individual factors, compared with wild-type ES cells, and then performed pair-wise correlation analysis of the log_2_-fold-changes for individual factors. Finally, the resulting Pearson correlation coefficients were subjected to hierarchical clustering to evaluate the similarity in the effects of depletion of individual factors.

### Genomic binding correlation analysis

We downloaded ChIP-sequencing data (BED files) of Oct4, Sox2, Nanog (GSE11431), Tip60 and p400 (GSE42329) from the GEO database. Using these data and our ChIP-sequencing data of Pontin, for each protein-coding gene (ENSEMBL NCBIM37), we evaluated Pontin binding enrichment ratio in its promoter region (2.5 kb-upstream and 500 b-downstream from the TSS)^68^ as the ratio of the total read counts that were normalized by their library size in the promoter region from Pontin- and GFP-IP (control) samples. We then performed correlation analysis of Pontin enrichment ratios in the promoter regions with enrichment ratios of the five factors (Oct4, Sox2, Nanog, Tip60 and p400) as previously described^69^. In brief, we sorted 22,073 protein-coding genes in a descending manner by their Pontin enrichment ratios, binned the sorted genes such that each bin includes 500 genes, and then computed the mean enrichment ratios of Pontin and the five factors for the genes in individual bins.

### Identification of differentially expressed lincRNAs

To analyse the expression of lincRNAs, we used the 226 multi-exonic lincRNAs that have been previously identified in mouse ES cells^18^. The lincRNAs with log_2_-fold-changes >0.58 (1.5-fold) were identified as differentially expressed lincRNAs.

### Assessment of protein coding probability of linc1253

We extracted the transcript sequences of protein coding genes and lincRNAs from ENSEMBL NCBIM37 database. Using the transcript sequences, we predicted the coding probabilities^46^ for total 3,099 lincRNAs and the same number of protein-coding genes randomly selected from the database and then generated the distribution of the coding probability. To predict the coding probability of linc1253, we used the transcript sequence of linc1253.

### Chromogenic RNA *in situ* hybridization

Detection of *linc1253* lincRNA in ES cells was performed using the RNAscope 2.0 Chromogenic Detection kit (Advanced Cell Diagnostics) in accordance with the manufacturer's instructions. Briefly, ES cells were fixed in 4% paraformaldehyde/PBS for 30 min and pretreated with 70% EtOH for 24 h. Fixed cells were hybridized with *linc1253*-specific probe designed from Advanced Cell Diagnostics based on *linc1253* sequence or negative control probe (DapB). Nuclei were stained with DAPI. Chromogenic signals and DAPI-stained images were detected with a Zeiss microscope

### RNA Immunoprecipitation (RIP) assay

The RNA immunoprecipitation protocol^48^ was adapted to analyse the interactions between Ezh2 and *linc1253* lincRNA. ES cells were crosslinked by 1% formaldehyde for 10 min at room temperature. The crosslinking reaction was stopped by addition of glycine (1 M, pH 7.0) to a final concentration of 0.25 M followed by incubation at room temperature for 5 min. The cells were washed with ice-cold PBS and resuspended in RIPA buffer (50 mM Tris-HCl, pH 7.4, 1% NP40, 0.5% sodium deoxycholate, 0.05% SDS, 1 mM EDTA and 150 mM NaCl) containing protease inhibitors and an RNase inhibitor. The cell suspension was sonicated and centrifuged for 10 min at 13,000 r.p.m., and the resulting supernatant was precleared by incubation with protein A-agarose beads. The precleared supernatant was incubated with IgG, anti-Ezh2, anti-Oct4 or anti-Pontin antibodies for 2 h at 4 °C. The beads were washed with RIPA buffer and resuspended with reversal buffer (50 mM Tris-HCl, pH 7.0, 5 mM EDTA, 10 mM DTT and 1% SDS) followed by incubation for 45 min at 70 °C to reverse the crosslinks. The immunoprecipitated RNAs were isolated according to the manufacturer's protocol (Invitrogen).

### Reprogramming

Reprogramming assay was done as previously described^70^. In brief, equal amounts of virus encoding different combination of factors were applied to 5 × 10^4^ plated Oct4-GFP transgenic MEFs. Oct4-GFP transgenic MEFs were reprogrammed by forced expression of retroviral Oct4, Sox2, and Klf4 (OSK). After 24 h, inactivated feeder cells were added and the culture was then maintained for up to 21 days. Reprogrammed cells were detected by GFP expression. The number of GFP-positive colonies was counted.

### Human Othologue of linc1253

We first blasted the *linc1253* gene sequence to the human genome and found a region to which two partial portions of the *linc1253* sequence were mapped. Second, since the blast analysis provided only the partial mapping of *linc1253*, near the partially mapped regions, we further identified other nine regions that share high sequence homologies with the *linc1253* sequence using UCSC genome browser. Thus, a potential *linc1253* location was identified as a region containing all the regions identified from both the blast and UCSC genome browser. Finally, mRNA-sequencing data (GSE16256) of human ES cells (H1 and H9 cells) revealed that no transcripts within this region were expressed.

### Statistical analysis

Statistical differences in test and control samples were determined by Student's *t*-test using the Statview package (Abacus Concepts, Berkeley, CA).

## Author contributions

K.B., J. B., D. H., H. L. and S.H.B designed the experiments. Y.J., K.K., I.-H.K. and H.L. are involved in generation, maintenance and analysis of *Pontin*-deficient mice and *Pontin*-depleted ES cells. J.K., H.-J.R.S., C.R.K. conducted molecular biology experiments. J.-E.P. and V.N.K. designed and conducted RNA-FISH experiments. K.B., J.B. and D.H. performed and analysed high-throughput sequencing data. K.B., H.J. and H.L. performed and analysed iPSC experiments. K.B., J.B., D.H., H.L. and S.H.B. wrote the manuscript.

## Additional information

**Accession codes:** The mRNA-sequencing and ChIP-sequencing data were deposited to the gene expression omnibus (GEO) database (Accession ID: GSE58206).

**How to cite this article:** Boo, K. *et al.* Pontin functions as an essential coactivator for Oct4-dependent lincRNA expression in mouse embryonic stem cells. *Nat. Commun.* 6:6810 doi: 10.1038/ncomms7810 (2015).

## Supplementary Material

Supplementary Information Supplementary Figures 1-5 and Supplementary Table 1

Supplementary Data 1 Summary of the alignment results for mRNA- and ChIP-sequencing data. Among the raw reads generated from Illumina Hiseq-2000 (WT, *Pontin*-depleted, and *Oct4*-depleted ES cells) and Hiseq-2500 (*linc1253* knockdown along with Mock ES cells, Pontin ChIP along with GFP ChIP) in the each sample, the reads after trimming low quality and adapter sequences were used (Used reads) for the alignment. On average, each 96.2 % and 94.3 % (Mapping rate) of the reads (Mapped reads) for mRNA- and ChIP-sequencing data were aligned to a reference mouse genome (Ensembl NCBIM37). Of the mapped reads, each 96.5 % and 70.3 % (Unique mapping rate) for mRNA- and ChIP-sequencing data were aligned to unique location (Uniquely mapped reads) in the genome. Exom Coverage denotes the fold of coverage of the mapped reads for the annotated exon region.

Supplementary Data 2 GOBPs represented by the genes affected by Pontin-depletion, Oct4-depletion, or linc1253 knockdown in ES cells. The GOBPs represented by the up-regulated genes in *Pontin-*depleted (**a**), *Oct4*-depleted (**c**), both *Pontin-* and *Oct4*-depleted (**e**), and *linc1253* knockdown ES cells (**g**). In parallel, the GOBPs represented by the down-regulated genes in *Pontin*-depleted (**b**), *Oct4*-depleted (**c**), both *Pontin-* and *Oct4-* depleted (**f**), and *linc1253* knockdown ES cells (**h**) are included. The count of the genes involved in each GOBP is shown. Finally, the GOBPs with *p*-value<0.1 (default cutoff in DAVID software) and Count>2 were determined as the ones significantly represented by the genes affected by Pontin or both Pontin and Oct4. Among the GOBPs, the colored GOBPs (**a**, **b**, **e**, and **f**) are representatives included in Figure 2b.

Supplementary Data 3 List of genes affected by *Pontin*-depletion, *Oct4*-depletion, and *linc1253* knockdown in ES cells. Up- and down-regulation of the DEGs in *Pontin-* or *Oct4*-depleted ES cells and their log_2_-fold-changes in the following comparisons are shown: (1 and 2) *Pontin*-depleted ES cells (OHT 3D or 4D) and (3) *Oct4*-depleted ES cells (Tc 2D) versus WT ES cells (a). Up- and down-regulation of the DEGs in *linc1253* knockdown ES cells and their log_2_-fold-changes are shown (b).

Supplementary Data 4 LincRNAs expressed in ES cells affected by *Pontin* or *Oct4* depletion . Up- and down-regulation of lincRNAs differentially expressed in *Pontin*-depleted (OHT 3D and 4D) or *Oct4*-depleted ES cells, compared to WT ES cells, and their log_2_-fold-changes are shown.
