# Supplementary material for: Pontin functions as an essential coactivator for Oct4-dependent lincRNA expression in mouse embryonic stem cells
Source: Nat Commun. 2015 Apr 10;6:6810. doi: 10.1038/ncomms7810 (PMC4403444; doi:10.1038/ncomms7810)
Supplement: Supplementary Information — Supplementary Figures 1-5 and Supplementary Table 1 [file ncomms7810-s1.pdf]

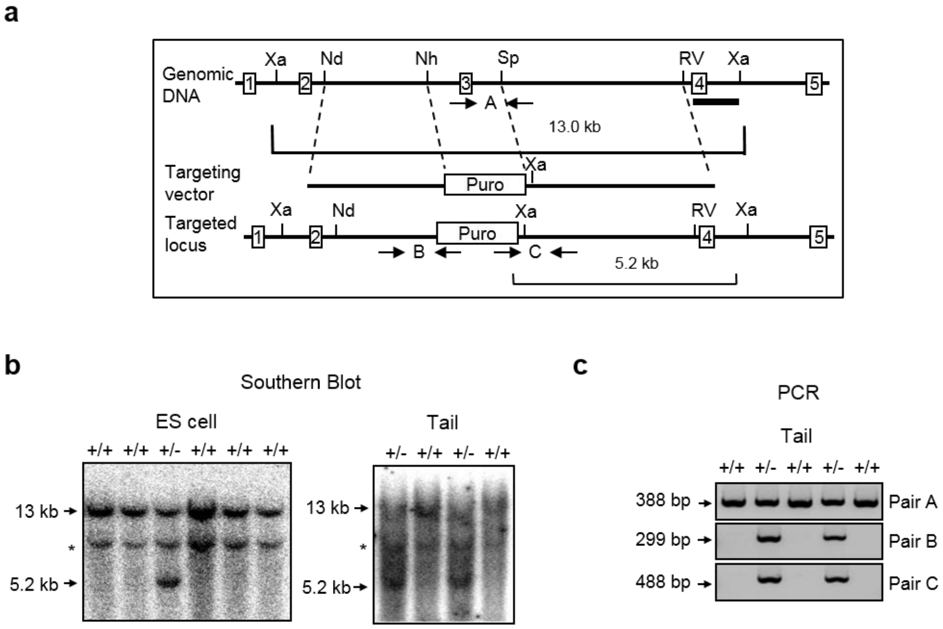

d

Age (dpc)	No. (%) of mice with indicated genotype			Total
	+/+	+/-	-/-	
Neonates	71 (42)	97 (58)	0 (0)	168
E10.5	8 (24)	25 (76)	0 (0)	33
E7.5	12 (27)	32 (73)	0 (0)	44
E3.5	14 (33)	22 (53)	6 (14)	42
Outgrowth	6 (18)	20 (59)	8 (23)	34

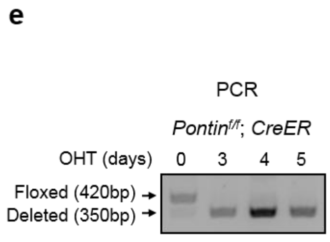

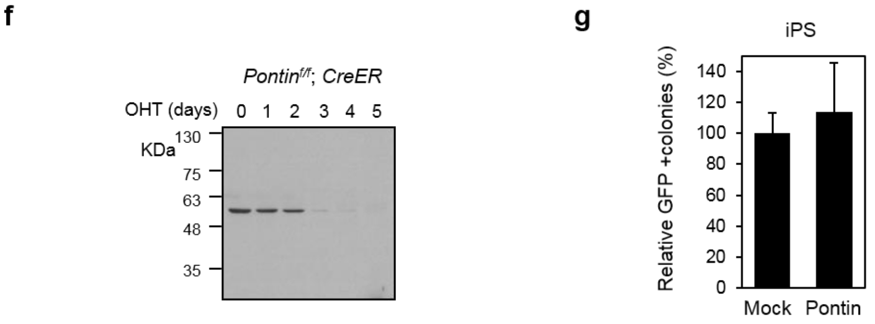

Supplementary Figure 1. *Pontin*-deficient mice are embryonic lethal

(**a**) Schematics of the mouse *Pontin* locus, targeting vector, and targeted allele after homologous recombination. Open boxes denote exons. The pgk-puromycin (Puro) resistance cassette replaces

exon 3. The position of 3’ probe is represented by black box. Xa, XbaI; Nd, NdeI; Nh, NheI; Sp, SpeI; RV, EcoRV. (**b**) Southern blot analysis to screen correctly targeted ES cells (left panel) or heterozygous mice (right panel). After XbaI digestion, the bands representing WT and mutant alleles were 13.0 kb and 5.2 kb, respectively. (**c**) PCR analyses with genomic DNAs extracted from tails of WT or heterozygous mice for genotyping. Tail DNAs were genotyped by PCR using the each pair of primers (indicated in panel **a**). (**d**) Genotypes of progeny from *Pontin*-heterozygous intercrosses. The numbers in parentheses refer to the percentage of each genotype per total embryos or neonates. (**e**) PCR analyses with genomic DNAs extracted from *Pontin*$^{f/f}$; *CreER* ES cells at 0, 3, 4 and 5 days after treatment with OHT. (**f**) Immunoblot analysis showing depletion of *Pontin* in *Pontin*$^{f/f}$; *CreER* ES cells at 1 to 5 days after treatment with OHT. There are no truncated forms of Pontin proteins. (**g**) An effect of Pontin overexpression on Reprogramming efficiency during OSK-mediated reprogramming of pOct4-MEFs to iPSCs.

Supplementary Figure 2

a

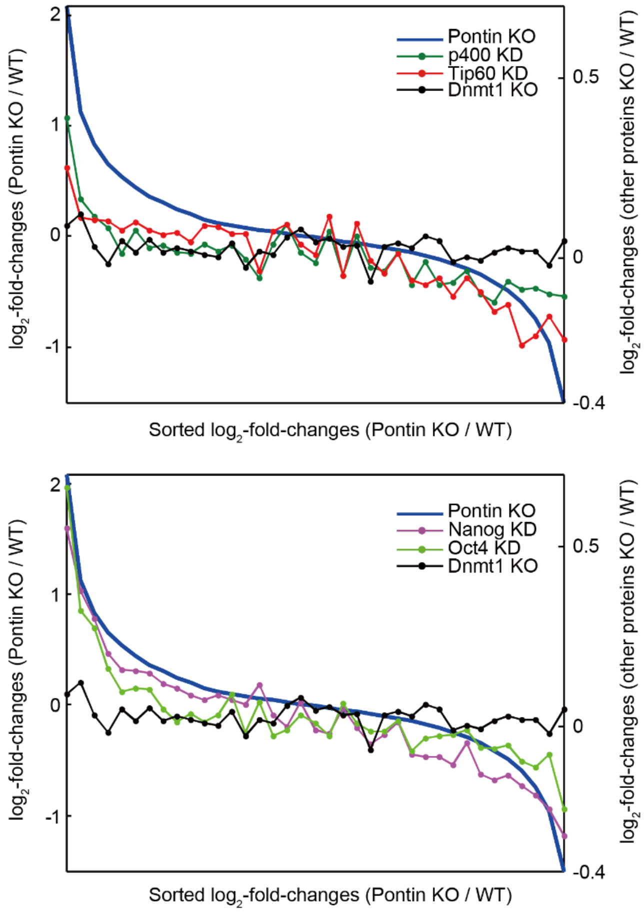

b

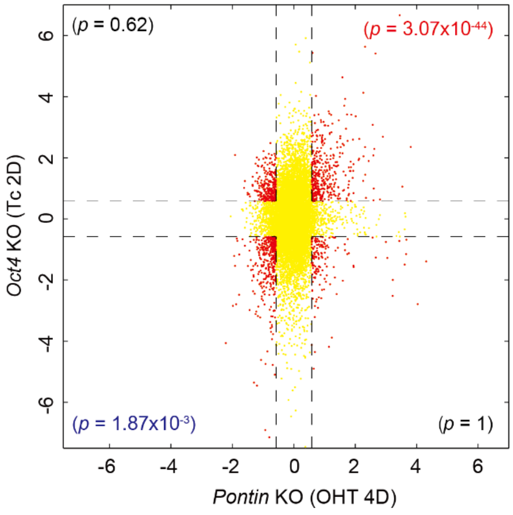

Supplementary Figure 2. Comparative expression analyses of *Oct4*- and *Pontin*-depleted ES cells

(**a**) Comparisons of log_2-fold-changes by Pontin depletion with those by Tip60, p400, Oct4, Nanog, and Dnmt1. X-axis indicates the sorted genes by log_2-fold-changes for Pontin, log_2(Pontin-depleted ES cells/WT ES cells). Left Y-axis indicates the mean log_2-fold-changes by Pontin depletion in individual bins of the sorted genes (400 genes/bin), and right Y-axis indicates the mean log_2-fold-changes by depletion of the other factors for the genes in the individual bins defined by Pontin. (**b**) Comparison of log_2-fold-changes in gene expression of *Pontin*-depleted ES cells at 4 days post-OHT treatment (OHT 4D) with those of *Oct4*-depleted ES cells at 2 days post-Tc treatment (Tc 2D). In the scatter plot, the red dots represent the DEGs (≥ 1.5-fold) between *Pontin*-depleted ES cells versus WT and *Oct4*-depleted ES cells versus WT. In each quadrant, the significance for the number of shared DEGs in *Pontin*- and *Oct4*-depleted ES cells, compared to WT ES cells, was computed by Fisher's exact test.

Supplementary Figure 3

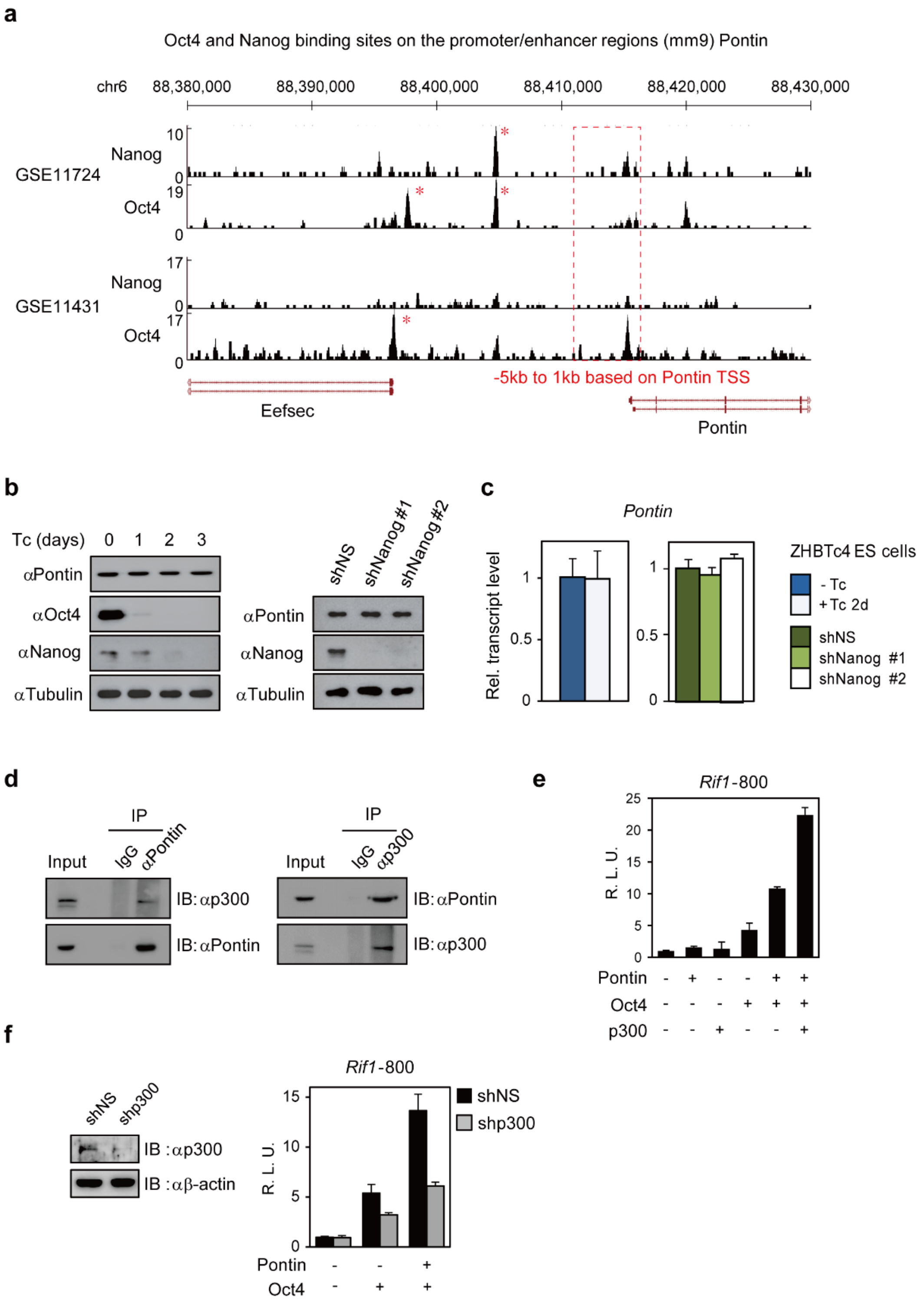

Supplementary Figure 3. Oct4 and Nanog do not influence the expression of Pontin

(**a**) Oct4 and Nanog binding sites in the promoter/enhancer region of Pontin. Two different sets of ChIP-sequencing data (GSE11431 and GSE11724) were used to explore the binding sites for Oct4 and Nanog. The binding sites of Oct4 and Nanog are represented as red asterisks. The inner red box indicates the genomic region between 5 kb upstream and 1 kb downstream from the TSS of Pontin. (**b**) Immunoblot analysis was performed using extracts from ZHBTc4 ES cells treated with Tc for 0, 1, 2 and 3 days (left panel) or infected with control or shNanog lentivirus (right panel). Indicated antibodies were used. (**c**) Quantitative RT-PCR analysis of Pontin with *Oct4*-depleted or *Nanog* knockdown ES cells. (**d**) Association of Pontin with p300 at endogenous expression level in ES cells by reciprocal co-immunoprecipitation assay. Immunoblot analysis using antibodies specific for the indicated proteins in ES cells was shown. (**e**, **f**) Luciferase assay was performed with reporters driven by *Rif1-800* promoter. Effects of p300 overexpression (**e**) or p300 knockdown (**f**) on *Rif1*-800 promoter-luciferase acitivity were shown in the presence of Pontin and Oct4. Luciferase activities were measured and normalized by β-galactosidase activity. Values are expressed as mean ± SD of three independent experiments.

Supplementary Figure 4

a

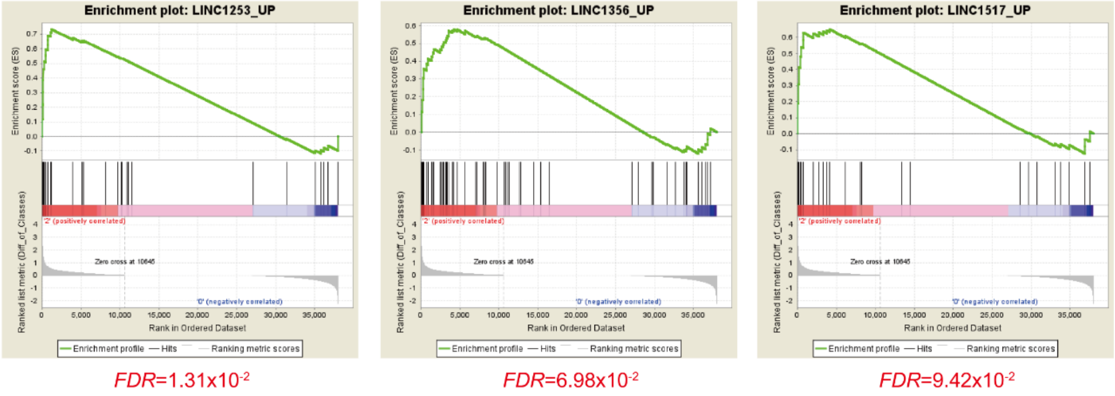

b

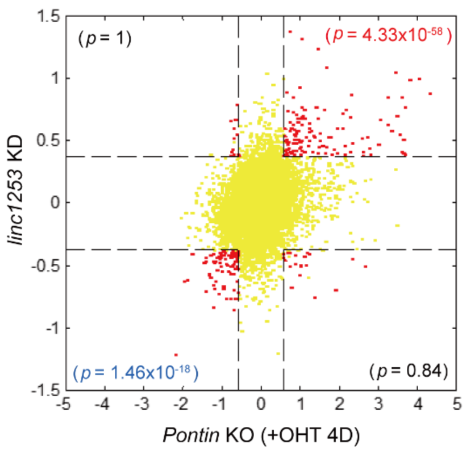

c

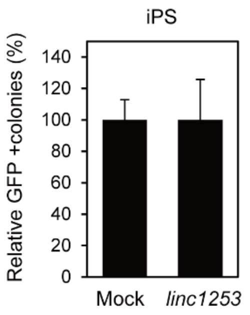

d

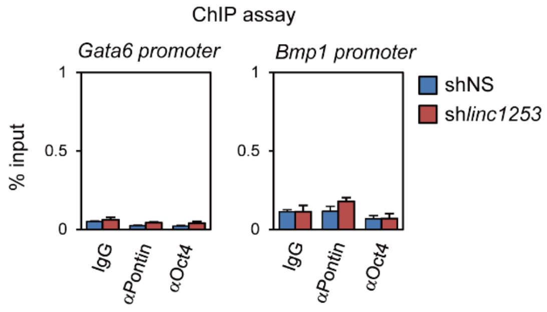

Supplementary Figure 4. Expression correlation between *Pontin*-depleted and *linc1253* knockdown ES cells

(**a**) Gene Set Enrichment Analysis (GSEA) between *Pontin*-depleted and *lincRNA* knockdown ES cells. For this analysis, we used a GSEA tool. This tool first ranked the genes based on their $\log_2$-fold-changes in the comparison of *Pontin*-depleted ES cells (OHT 4D) versus WT. In the enrichment plot, the histogram of $\log_2$-fold-changes for the ranked genes is shown at the bottom, and the gradient of $\log_2$-fold-changes is shown in the color bar in the middle (up-regulated genes were denoted as 'positively correlated' while down-regulated genes were denoted as 'negatively correlated' in *Pontin*-depleted ES cells). For each lincRNA, we obtained the up-regulated genes by knockdown of the *lincRNA* in ES cells previously reported. Based on the rank correlation of the up-regulated genes between the *lincRNA* knockdown and *Pontin*-depleted ES cells, the GSEA tool computes the enrichment score profile and then the false discovery rate (FDR) representing the significance of the enrichment. Of the 16 lincRNAs co-regulated by both Pontin and Oct4, 5 lincRNAs (linc1253, linc1356, linc1517, linc1562, and linc1602) have been shown to function as repressors of lineage differentiation program in ES cells. Among the 5 lincRNAs, 3 lincRNAs (linc1253, linc1356, and linc1517) with FDR<0.1 whose knockdown led to significant shared changes by *Pontin* depletion in ES cells were shown here. (**b**) Comparison of $\log_2$-fold-changes in gene expression of *Pontin*-depleted ES cells (OHT 4D) with those of *linc1253* knockdown ES cells. In the scatter plot, the red dots represent the DEGs in the comparisons of *Pontin*-depleted ES cells versus WT and *linc1253* knockdown ES cells versus WT. In each quadrant, the significance for the number of shared DEGs in *Pontin*-depleted and *linc1253* knockdown ES cells, compared to WT ES cells, was computed by Fisher's exact test. (**c**) An effect of *linc1253* overexpression on Reprogramming efficiency during OSK-mediated

reprogramming of pOct4-MEFs to iPSCs. (**d**) ChIP assays were performed on indicated gene loci for control IgG, anti-Pontin and anti-Oct4 antibodies in ES cells infected with shNS (black bars) or sh*linc1253* (gray bars) lentiviruses. Neither Pontin nor Oct4 is recruited to these gene loci. Values are expressed as mean ± SD of three independent experiments. *p<0.05.

Supplementary Figure 5. Original images of the cropped blots shown in the paper

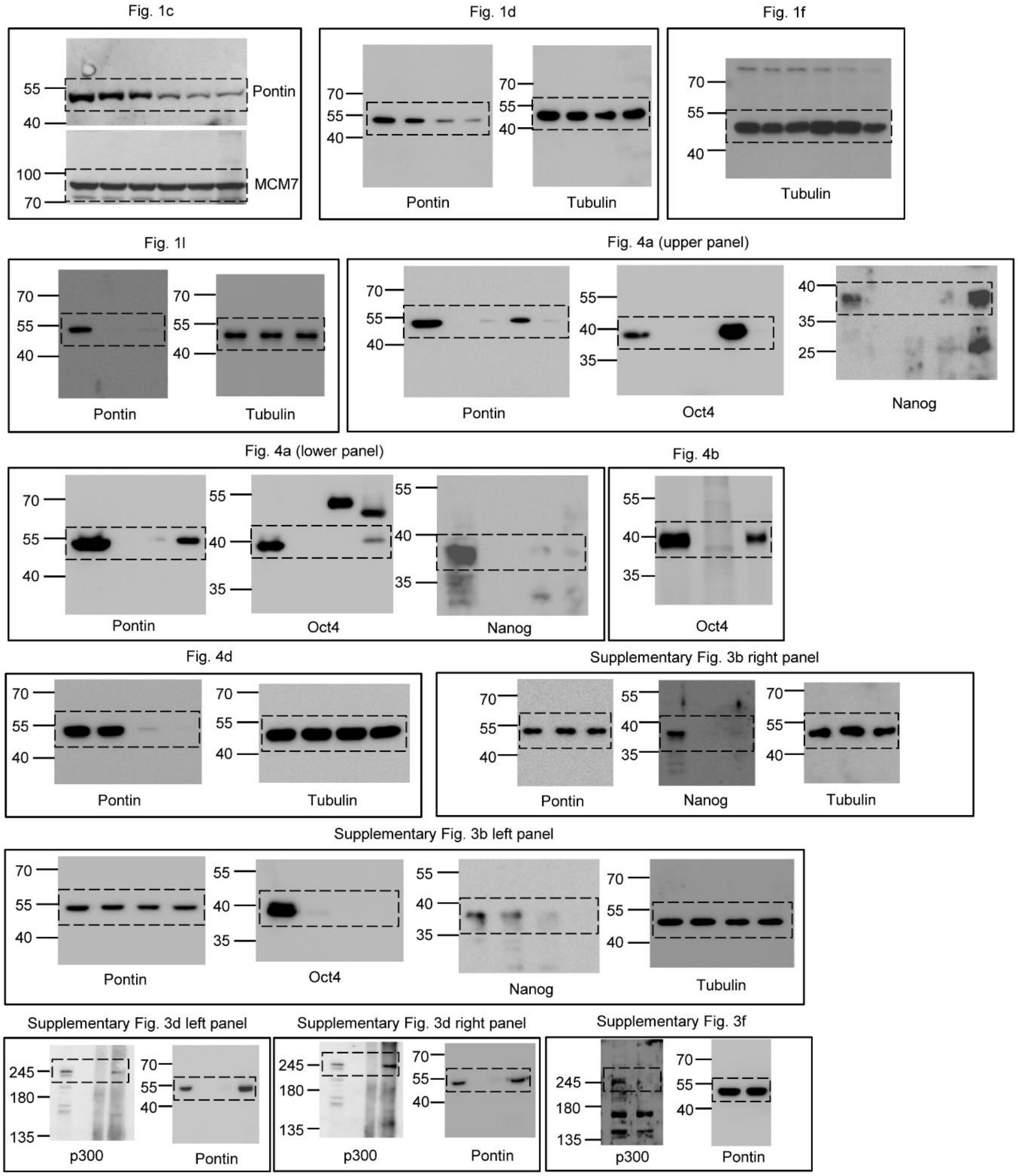

Supplementary Table 1. List of antibodies used in the study

Antibody	Vendor/ Source	Catalog number	IB	IF	IP	ChIP	RIP	FACS
anti-Oct4 (C-10)	Santa Cruz	sc-5279	1/1000	1/100	2 μg	1 μg	1 μg	
anti-Pontin (SAB4200194)	Sigma-Aldrich	SAB4200194	1/1000	1/100	2 μg	8 μg	1 μg	
anti-Nanog	abcam	Ab21624	1/1000		2 μg	1 μg		
anti-Ezh2	BD bioscience	#621667				1 μg	1 μg	
anti-histone H3K27Ac	abcam	ab4729				0.5 μg		
anti-p300 (C-20)	Santa Cruz	sc-585	1/1000		2 μg	2 μg		
anti-p300 (N-15)	Santa Cruz	sc-584				2 μg		
anti-p300 (H-272)	Santa Cruz	sc-8981				2 μg		
anti-H3	Cell Signaling Technology	#2650				1 μg		
anti-SSEA-1	R&D systems	MAB2155		1/100				
anti-BrdU	Developmental Studies Hybridoma Bank	G3G4						1/100
Alex Fluor 488 goat anti-mouse	Invitrogen	A-11001						1/100

IB, Immunoblot; IF, Immunofluorscence; IP, Immunoprecipitation; ChIP, ChromatinIP; RIP, RNA IP; FACS, Fluorescence-activated cell sortingS
